# Regulation of Parkinson’s disease-associated genes by Pumilio proteins and microRNAs in SH-SY5Y neuronal cells

**DOI:** 10.1371/journal.pone.0275235

**Published:** 2022-09-29

**Authors:** Lisa J. Snoderly-Foster, Wendy M. Olivas

**Affiliations:** Department of Biology, University of Missouri-St. Louis, St. Louis, Missouri, United States of America; University of Surrey, UNITED KINGDOM

## Abstract

Parkinson’s disease is the second most common age-related, neurodegenerative disease. A small collection of genes has been linked to Parkinson’s disease including *LRRK2*, *SAT1*, and *SNCA*, the latter of which encodes the protein alpha-synuclein that aggregates in Lewy bodies as a hallmark of the disease. Overexpression of even wild-type versions of these genes can lead to pathogenesis, yet the regulatory mechanisms that control protein production of the genes are not fully understood. Pumilio proteins belong to the highly conserved PUF family of eukaryotic RNA-binding proteins that post-transcriptionally regulate gene expression through binding conserved motifs in the 3’ untranslated region (UTR) of mRNA targets known as PUF Recognition Elements (PREs). The 3’UTRs of *LRRK2*, *SNCA* and *SAT1* each contain multiple putative PREs. Knockdown (KD) of the two human Pumilio homologs (Pumilio 1 and Pumilio 2) in a neurodegenerative model cell line, SH-SY5Y, resulted in increased *SNCA* and *LRRK2* mRNA, as well as alpha-synuclein levels, suggesting these genes are normally repressed by the Pumilio proteins. Some studies have indicated a relationship between Pumilio and microRNA activities on the same target, especially when their binding sites are close together. *LRRK2*, *SNCA*, and *SAT1* each contain several putative microRNA-binding sites within the 3’UTR, some of which reside near PREs. Small RNA-seq and microRNA qPCR assays were performed in both wild type and Pumilio KD SH-SY5Y cells to analyze global and differential microRNA expression. One thousand four hundred and four microRNAs were detected across wild type and Pumilio KD cells. Twenty-one microRNAs were differentially expressed between treatments, six of which were previously established to be altered in Parkinson’s disease patient samples or research models. Expression of ten miRs predicted to target *LRRK2* and *SNCA* was verified by RT-qPCR. Collectively, our results demonstrate that Pumilios and microRNAs play a multi-faceted role in regulating Parkinson’s disease-associated genes.

## Introduction

As quality of life and life expectancy increase, age-related diseases are demanding more attention, especially those of neurodegenerative etiology. Parkinson’s Disease (PD) is one of the most significant age-related neurodegenerative diseases, second only to Alzheimer’s Disease [1]. Approximately 500,000 Americans are living with PD, as many as 500,000 more may be mis- or undiagnosed, and the number of diagnosed is anticipated to double by 2040 [2]. This trend has generated the need for more research into the molecular causes of disease onset and progression.

PD clinical symptoms are linked to a loss of neurons within the substantia nigra (SN) of the midbrain involved in the patterning and control of voluntary muscle movements [3–5]. The neurons of the substantia nigra pars compacta (SNpc) are catecholaminergic, producing large amounts of the neurotransmitter dopamine. To best study the molecular mechanisms of PD in a laboratory setting, a model system that closely resembles/mimics the chemical profile of SNpc neurons is needed. SH-SY5Y is a human neuroblastoma cell line that produces almost exclusively neuron-like cells that retain dopamine-β-hydroxylase activity but have diminished activity of the other catecholamines [6,7]. Thus, SH-SY5Y cells provide a better morphological and physiological mimic of SN cells than other non-neural human or neural non-human cell lines and are widely used in PD, Alzheimer’s disease, and Amyotrophic Lateral Sclerosis research [6].

Two key genes linked to PD pathogenesis are *SNCA* and *LRRK2*. The synuclein alpha (*SNCA*) gene encodes the protein alpha-synuclein (α-syn) that is found aggregated in the Lewy bodies associated with PD histopathology [4,5,8–10]. Mutations associated with dominant familial and sporadic PD are prevalent in the Leucine-Rich Repeat Kinase 2 (*LRRK2*) gene, also distinguished as PARK8 or Dardarin [5,11–13]. The relationship between α-syn and LRRK2 function in PD pathogenesis is complex and our understanding incomplete; however, overexpression and mutations in these genes have been linked to neuronal death and cytotoxicity [3,4,14–17]. Overexpression of α-syn resulting from multiplication of the *SNCA* locus is linked to earlier onset of autosomal dominant PD, thus α-syn abundance is a factor in pathogenesis [15]. Mutations in *SNCA* also lead to increased α-syn protofibrillization, and dopamine appears to play a role in the stabilization of these insoluble α-syn fibers that comprise a large portion of Lewy bodies [10,14,18]. Mutations in *LRRK2* result in increased target phosphorylation, formation of LRRK2-containing proteinaceous inclusions, α-syn aggregation and toxicity, and/or death of dopaminergic neurons of the SNpc [16,17,19,20]. Clinically relevant mutations within coding regions of PD genes have almost exclusively been investigated. However, mutations identified outside of the coding regions are bringing to light the relevance of the untranslated regions (UTRs), which possess critical elements for post-transcriptional regulation of mRNA decay and translation, and the potential to impact disease development. For example, a single nucleotide (nt) polymorphism located within the 3’UTR of *SNCA* and a 3nt deletion within the 3’UTR of *SAT1*, a gene encoding a polyamine pathway protein linked to α-syn toxicity, have been confirmed as clinically significant mutations [21,22]. The *SAT1* deletion falls within a motif that strongly resembles a Pumilio Response Element.

PUF proteins are an evolutionarily conserved family of eukaryotic RNA-binding proteins (RBPs) that post-transcriptionally downregulate expression of bound target mRNAs by promoting rapid mRNA decay and/or inhibiting translation [23,24]. PUFs are named for the first two discovered family members, the Pumilio protein (PUM) of *D*. *melanogaster* and the fem-3-binding factor (FBF) proteins of *C*. *elegans* [24,25]. PUF family proteins play a vital role in embryogenic patterning and sex cell development in these species [23,26–29]. In Drosophila, Pumilios also regulate neuronal excitability, dendritic length and branching, neuromuscular junction morphology and synaptic function, as well as establishment of long-term memory [30–33].

The human genome encodes two PUF protein homologs called Pumilio 1 (PUM1) and Pumilio 2 (PUM2) [23,34]. A common characteristic among PUF proteins is the presence of the PUF homology domain, a tandemly repeating region of amino acid sequence that binds to RNA at AU-rich motifs called PUF Recognition Elements, also referred to as Pumilio Response Elements (PREs) [24,25,34–36]. Since we are specifically reporting on human PUF proteins in this study, we will henceforth use the term PUM to refer to these proteins, and PRE will represent the human PUM Response Element. PREs are typically located within the 3’UTR of target transcripts but are sometimes found in the 5’UTR and coding region [34,37]. The PRE length is variable, however a core motif consisting of eight nts is highly conserved [23,34]. In humans, the optimal PRE sequence is UGUA(A/U)AUA, and the presence of a PRE is a strong predictor of a PUM protein target [34,38,39]. We have identified numerous putative PREs within the 3’UTRs of *SAT1*, *SNCA*, and *LRRK2*.

Experimental validation and clustering of target mRNAs by gene ontology reveal the role of mammalian PUMs in a broad range of cellular processes, including neuronal morphology and function [34,37]. Studies using mice models and mammalian cell lines have also confirmed the role of PUMs, especially PUM2, in synaptic activity at the neuromuscular junction, neurogenesis, dendritic morphogenesis, learning and memory [40–43]. In mice, PUM1 directly regulates *ATXN1* levels. *ATXN1* overaccumulation has been implicated in the development of spinocerebellar ataxia type 1, a disease in which cerebellar neurodegeneration results in a loss of motor coordination. In humans and mice, mutations that reduce or eliminate PUM1 levels lead to developmental delays, seizures, loss of cerebellar Purkinje cells, and progressive motor dysfunction [44,45].

The potential relationship between human PUM proteins and neurodegenerative diseases has been implicated, but not yet fully explored. Other RBPs are also involved in several neurodegenerative disorders [46]. Changes in abundance and targeting capacity of RBPs, as well as RBP-induced protein misfolding and aggregation have been associated with neuronal dysregulation [46,47]. DJ-1, HuD, and TTP are RBPs that have been found to specifically influence PD by modulating miRs associated with neuronal function, proliferation, and differentiation [47]. Global studies to identify targets of human PUM proteins have reported PUM binding to *SNCA* mRNA and increased expression of *SNCA* in PUM-deficient conditions; however, these studies found no relationship between PUMs and *LRRK2* [34,37,48]. Moreover, these studies were carried out in HEK293 and HeLa cell lines, not in a neurodegenerative disease model line, and are not in agreement about the significance of *SNCA* differential expression [34,37,48]. Based on the presence of high confidence PUM binding elements in the 3’UTRs of *SNCA*, *LRRK2*, and *SAT1*, we hypothesized that mRNA decay/translation of these genes is regulated by PUM proteins. In this study, we explore the role of PUM proteins in the regulation of multiple PD genes using the model neuronal human cell line SH-SY5Y. We have determined that knockdown of cellular PUM1 and PUM2 levels in SH-SY5Y cells results in increased expression of *SNCA* and *LRRK2*, suggesting that PUMs play a role in the post-transcriptional regulation of these genes. We also confirm direct PUM regulation of *SNCA* through mutational analysis.

In addition to regulation by PUMs, the 3’UTRs of *SNCA*, *LRRK2*, and *SAT1* contain predicted microRNA (miR) binding sites. MiRs are short, non-coding RNAs that post-transcriptionally regulate mRNAs [49]. Evidence to support a connection between PD and miRs is plentiful. Attenuation of the miR biogenesis pathway through knockdown of *Dicer* results in a loss of DA neurons [50,51]. Directed ablation of Dicer in mouse midbrain dopaminergic neurons results in neuronal death and a PD-like phenotype [52–54]. Additionally, miR profiles have been found to be altered in serum and cerebrospinal fluid samples from PD patients [55–57]. A few predicted miRs, such as miR-7, miR-153, and miR-205, have been experimentally confirmed as negative regulators of *SNCA* and *LRRK2* expression [50,58,59]. While PUMs and miRs can each directly regulate mRNA targets, previous studies also suggest a relationship between PUMs and miRs. Galgano *et al*. discovered the enriched presence of high confidence predicted miR sites in PUM targets and a significant proximal relationship between miR sites and PRE motifs [34]. Messenger RNAs with PREs within 50nts of binding sites for expressed miRs decay faster than transcripts with distantly spaced motifs [60]. Computational analyses indicate that PUM binding may induce mRNA secondary structure changes that affect accessibility of miR binding sites, suggesting that miR activity close to PREs may be modulated by PUMs [34,60,61]. In the p27 3’UTR, the binding of PUM1 induces a structural shift that promotes accessibility of the nearby miR-221/222 site, providing empirical evidence to support cooperativity between PUMs and miR [62]. In this study, we performed small RNA-seq (sRNA-seq) to establish which miRs predicted to bind PD genes are expressed in SH-SY5Y cells. Moreover, since the regulation of PD-associated genes by miR may be related to their regulation by PUM proteins, we also generated a global miR profile for PUM1/PUM2-deficient SH-SY5Y cells to search for differential expression of miRs linked to PUM activity. Twenty-one differentially expressed miRs (DEMs) were identified, six of which are predicted to target the 3’UTRs of PD-associated genes *LRRK2* and *SNCA*. Our results suggest that protein levels of PD-associated genes hinge upon the balance of activities of constituents within a complex regulatory network that includes PUM proteins and miRs.

## Materials and methods

### Cell culture and transfections

All tests were performed in the PD model cell line SH-SY5Y (ATCC CRL-2266). Cells were maintained in culture at 37°C under 5% atmospheric CO_2_ in growth media containing Eagle’s Minimal Essential Media (ATCC 30–2003) and Hamm’s F12 nutrient mixture (Thermo Fisher 31765035) at 1:1, with 10% fetal bovine serum (uncharacterized, denatured) and 5% Penicillin/Streptomycin. Cells were grown in 75 cm^2^ flasks and passaged at approximately 70% confluency (every 10–14 days). Transfections were performed at time of passage. Cells were harvested by trypsinization, washed in sterile 1X PBS and counted with a cellometer (Nexcelom Biosciences). SiRNAs were transfected into cells by electroporation with the Neon Electroporation System (Thermofisher). Cells were electroporated at 1200 V for 20 ms/pulse x 3 pulses. Electroporated cells were recovered in growth media without antibiotics.

### RNA isolation

RNA was extracted 48 hours post-transfection. Cells were liberated from the flask through trypsin treatment and aspirated into a conical tube. Cells were washed twice with sterile 1X PBS and pelleted, then lysed for RNA purification using the Qiagen RNeasy Mini Kit (74106) according to the manufacturer’s protocol. To eliminate any residual genomic DNA, RNA samples were DNase I-treated using the Turbo DNA-free kit (Ambion AM1907). RNA quantification and purity was assessed using a NanoPhotometer.

### RNA interference and RT-qPCR

PUM expression was knocked down using Silencer Select siRNAs that target PUM1 (Ambion 4392420; assay ID: s18682) and PUM2 (Ambion 4392420; assay ID: s23671). Non-targeting siRNA was used as a negative control (Ambion 4390843). siRNAs were transfected at 250 nM in accordance with manufacturer protocols. Cells were harvested 48 hours post-transfection, pelleted, and washed twice in sterile 1X PBS. RNA was extracted and purified as described in the RNA isolation section. 75 ng of purified RNA was converted to cDNA using an iScript RT Supermix for RT-qPCR kit (BioRad 1708841). Diluted cDNA (20x), 375 nM primers designed for qPCR (S1 Table), and SSO Advanced Universal SYBR Green Supermix (BioRad 1725274) were mixed and aliquoted into white Real-Time PCR 96-Well Microplates. Quantitative PCR was performed on a CFX96 Real-Time PCR detection system (BioRad) for 39 cycles. All tests were performed in a minimum of biological and technical triplicate. Data was analyzed using CFX software and differences represented in figures as Log2 transformed average relative fold change (2^-ΔΔCT^). Variation is calculated as SEM.

### Western blotting

Proteins were extracted 72 hours post-transfection. Cells were harvested, washed twice in sterile 1X PBS, and pelleted. Cells were lysed using RIPA buffer (Thermo Scientific 89900) with HALT protease inhibitor (Thermo Scientific 78430). Protein concentrations within whole cell lysates were quantified in a BCA assay using Pierce BCA reagents (Thermo Scientific 23227) and standards (Thermo Scientific 23208). 10 μg of protein was mixed with 6X load buffer (SDS-based Laemmli formulation), heated for 5 minutes at 100°C, and loaded onto Bis-Tris gels. Larger proteins were separated on 7.5% gels containing acrylamide/bis-acrylamide at 37.5:1 (2.7% crosslinker) and electrophoresis performed at 125 mV using MOPS-based run buffer. Smaller proteins were separated on 10% gels containing acrylamide/bis-acrylamide at 29:1 (3.3% crosslinker) and electrophoresis performed at 125 mV using an MES-based run buffer. Since the predicted size of LRRK2 is larger than 250 kDa and α-syn is only 14 kDa, Spectra Multicolor High Range protein ladder (Thermo Scientific 26625) and PageRuler protein ladder (Thermo Scientific 26617) were used as standards, respectively. Separated proteins were transferred to nitrocellulose membranes using the BioRad Mini-Protean Tetra Blotting module for a wet tank transfer protocol. Transfers were performed at 30 V/300 mAmps for 16 hours. Membranes were equilibrated in 1X PBS-T and then incubated with blocking solution (1X PBS-T + 5% milk) for 1.5 hours at room temp. Membranes were washed 2x with 1X PBS-T prior to incubating for 1 hr at room temp with the primary antibody (diluted in blocking solution). Membranes were washed 5x for 3 minutes each with 1X PBS-T before and after incubating with the secondary antibody (diluted in 1X PBS-T). Antibodies are listed in S2 Table. Protein banding was visualized using Enhance Chemiluminescence (Thermo Scientific 34075) reagents. The luminescent signal was detected/captured using a BioRad ChemiDoc Imaging System. Annotated raw blots can be viewed in S1 Fig. Band intensities were quantified using Image Lab Software (BioRad). Intensities were normalized to beta tubulin (TUBB) levels. Sample variation was calculated as SEM. Western blotting was performed in a minimum of biological triplicate.

### Luciferase assays

The 3’UTR of *SNCA* was cloned into a psiCHECK-1 vector (Promega C8011) downstream of a synthetic *Renilla* luciferase gene. The core TGTA of the *SNCA* 3’UTR canonical PRE site in the reporter was mutated to ACAC with the QuickChange XL Site-Directed Mutagenesis kit from Agilent Technologies (catalog # 200516). The mutagenesis primer sequences are reported in S1 Table. At time of cell culture passage, SH-SY5Y cells were harvested, washed in 1X PBS, and transfected with luciferase reporters by electroporation. Each well of a white, flat bottom, tissue culture-treated, 96-well plate received 50,000 cells transfected with 1.25 μg of reporter plasmid (wild type or mutated *SNCA* 3’UTR) and a firefly luciferase-expressing co-reporter (pGL4.13; Promega E6681) at a 25:1 reporter to co-reporter ratio. At 48 hrs post-transfection, luciferase activity was measured with the Dual-Glo Luciferase Assay System (Promega E2940) on a Perkin Elmer Victor3 plate reader. Relative light units were measured for *Renilla*, then firefly, luminescence as counts per second. Measurement of each well was repeated in triplicate and values averaged. Background was measured as cells transfected with no reporters. Background was subtracted from each well and the *Renilla*/firefly relative response ratio was calculated and used to determine relative expression levels. Assays were performed in biological and technical triplicate. Sample variation was reported as SEM.

### Library preparation for small RNA sequencing

RNA qualification and quantification, library preparation, and sequencing was performed by the Experimental Department of Novogene Genomics. Prior to library prep, RNA was electrophoresed on a 1% agarose gel to ensure samples were not degraded or contaminated. Purity was determined using a NanoPhotometer. Integrity and quantification of RNA was determined using the Agilent RNA Nano 6000 Assay Kit. Six samples were sequenced, for a total of 3 biological replicates per treatment. Three μg of each RNA sample, with a purity of >1.95, was used for small RNA library preparation. Libraries were generated using NEBNext Multiplex Small RNA Library Prep Set for Illumina (New England Biolabs) according to manufacturer’s protocols. Index codes were added to link sequence data back to the original sample. Library quality was assessed using the Agilent Bioanalyzer 2100 System. Indexed samples were clustered using a TruSeq SR Cluster Kit v3-cBot-HS (Illumina) according to manufacturer’s protocol. Fifty bp single-ended reads were generated from sequencing of clustered libraries on an Illumina Novaseq 6000 platform with a sequence depth of ≥10 million read pairs per sample. Raw data is deposited as FASTQ files in the NCBI SRA under BioProject ID PRJNA836782.

### MicroRNA expression analysis

Data analysis was performed by the Gene Regulation Department of Novogene Genomics. Raw reads in FASTQ format were processed using custom perl and python scripts. To obtain clean reads, reads containing poly-N, 5’ adapter contamination, a lack of 3’ adapter or insert tag, and low quality reads were removed from the raw data. The small RNA tags were mapped to the human genome (genome ID: ensembl_homo_sapiens_grch38_p12_gca_000001405_27) by Bowtie without mismatch for analysis of expression and distribution on the genome [63]. The mapped small RNA tags were used to search for and identify known miRs using miRBase20.0, mirdeep2, and srna-tools-cli. Custom scripts were used to analyze miR counts, base bias at the first position, and base bias at each position of identified miRs. Small RNA tags were submitted to RepeatMasker, Rfam database, and other species-specific databases to remove tags originating from protein-coding genes, repeat sequences, rRNA, tRNA, snRNA and snoRNA. Novel miRs were predicted by submitting miR precursor secondary structure to miREvo and mirdeep2 [64,65]. MiR expression levels were estimated by TPM (transcript per million) as in Zhou et al., 2010 [66]. Mapped read count is divided by the total reads multiplied by 1 million to obtain normalized expression. Based on the example of previous studies, we set the threshold for expression of miRs as 10 TPM in 2 out of the 3 wild type (WT) samples [67,68]. Raw counts and counts normalized to TPM for miRs that had at least 1 read in any sample are listed in S3 Table. Differential Expression between WT and PUM knockdown (KD) samples was evaluated using DESeq R package (1.8.3). Prior to determining which miRs are differentially expressed, raw reads were normalized using the mean of ratios method to account for variability between samples that may result from technical factors, such as library size and composition. The geometric mean of each gene was used to create size-based, sample-specific scaling factors for read normalization. The mean of newly normalized reads was then plotted against variance to determine dispersal. MiRs considered outliers during dispersal analysis or linear modeling, meaning variability between samples for that miR cannot be effectively adjusted for, were removed from later analysis [69]. The miRs that passed the filtering stages of differential analysis were used to distinguish significantly differentially expressed miRs. The p-value was adjusted using the Benjamini and Hochberg method. A corrected p-value of 0.05 was used to determine significant differential expression (S4 Table).

### Mature microRNA quantification assays

Levels of mature miRs were quantified in WT and PUM 1/2 KD cellular backgrounds. Fifty ng of total RNA purified with Qiagen RNeasy Mini Kit was used as input for a 10 μL cDNA synthesis reaction using the Qiagen miRCURY LNA RT kit (339340). UniSp6 spike-in template was added to the RT reaction to serve as an exogenous control for RT and PCR amplification efficiency. The cDNA was diluted 24X for all targets and 4 μL used per well of a 10 μL qPCR reaction. The only exceptions were miR-205-5p and miR-200a-3p, which used 200ng total RNA input and a 7X dilution of cDNA. Quantitative PCR was performed using Qiagen miRCURY LNA SYBR Green PCR kit (339346) and specific miRCURY LNA miR PCR Assay primer pairs (339306). The qPCR protocol, as outlined in the SYBR Green kit manual, was performed on a BioRad CFX machine. MiR levels were normalized to *SNORD38B*. Five candidate reference genes were assessed: *SNORD38B*, *SNORD44*, *RNU5G*, 5S *rRNA*, and *U6*. *SNORD38B* demonstrated the most stable expression across multiple biological replicates with the least variation between treatments and was therefore used as the endogenous control for normalization (S2 Fig).

### Statistical analysis

Differences in expression of *LRRK2*, *SNCA*/ α-syn, and *SAT1* at the mRNA and proteins levels from RT-qPCR and Western Blotting, respectively, as well as miR levels from PCR assays and luciferase activity from luciferase assays, were analyzed using a paired, two-tailed Student’s T-test. Data transformations and statistical analyses were conducted in GraphPad Prism 9 software.

## Results

### PUM Response Elements predicted in the 3’UTRs of *SNCA*, *LRRK2*, and *SAT1*

**Fig 1:**
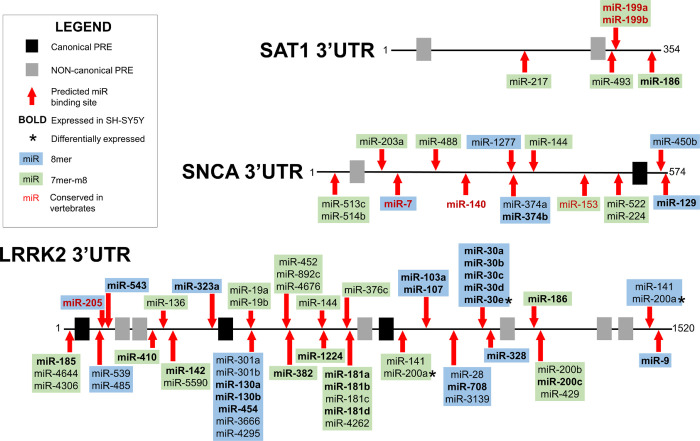
PRE and microRNA binding sites in the 3’UTRs of *LRRK2*, *SNCA*, and *SAT1*. The black lines are a linear representation of the 3’UTRs of *SAT1*, *SNCA*, and *LRRK2* transcripts. The number 1 indicates the nt immediately following the stop codon (5’ end of the 3’UTR). 3’UTR lengths for *LRRK2* and *SAT1* were based on transcript data from NCBI and 3’UTR length for *SNCA* was based on NCBI data as well as empirical data from expressed sequence tags [22]. Motifs with the sequence UGUA(A/U/C)AUA are considered canonical PREs and their locations on the 3’UTRs are represented by black boxes. Motifs that do not match the canonical sequence but contain a UGUA with downstream AU-rich region are considered non-canonical and are represented by gray boxes. The relative locations of predicted miR-binding sites are represented by red arrows. MiRs confirmed to be expressed in SH-SY5Y cells using sRNA-seq are bolded. MiRs with an asterisk (*) were determined to be differentially expressed based on DEseq2 analysis. MiR family conservation is based on TargetScanHuman 8.0 categorizations [71]. Broadly conserved miR families, those conserved among vertebrates, are represented by red text. All other levels of conservation are represented by black text. 8mer sites are highlighted in blue and 7mer-m8 sites are highlighted in green. 3’UTRs and predicted sites are not drawn to scale.

Having first identified a clinically relevant mutation in a potential PRE within the 3’UTR of *SAT1* [21], we wished to determine if other Parkinson’s Disease-associated genes contained PRE sites in their 3’UTRs. The PRE for human PUM1 and PUM2 proteins is AU-rich with a canonical eight nt core motif of UGUA(A/U)AUA. However, PUF proteins are often flexible in their binding to target sequences providing the UGUA remains intact, since downstream bases within the AU-rich region can be flipped out that do not match the binding surface [70]. Thus, motifs that are not an exact match to the canonical sequence but contain the UGUA and a downstream AU-rich region may retain function and are considered non-canonical. The 3’UTR sequences from NCBI for human *SAT1* (NM_002970.4), *SNCA* (NM_000345.3) and *LRRK2* (NM_198578.4) were thus searched for both canonical and non-canonical PRE motifs. Putative functional sites were chosen based on the level of identity with the canonical PRE motif, including a required presence of the core UGUA and the presence of a downstream AUA (or AU-rich region). Canonical PREs were found in the 3’UTRs of *SNCA* and *LRRK2* (Figs 1 and S3). A single non-canonical site was also identified in *SNCA*, and multiple non-canonical sites reside in the *LRRK2* 3’UTR. *SAT1* does not contain a canonical binding motif but has two non-canonical sites (Fig 1). When the PRE sequences within the 3’UTRs of *SNCA*, *LRRK2*, and *SAT1* are compared across 13 mammalian species, including humans, conservation can be observed (S4 Fig). The putative PRE sites in *SNCA* and *SAT1*, both canonical and non-canonical, exhibit strong conservation. Cross-species conservation of sequence is also strong at *LRRK2* canonical sites. The *LRRK2* non-canonical sites demonstrate less overall conservation and PREs closer to the 5’ end of the 3’UTR retain more sequence similarity across species (S4 Fig). The conservation of PRE sequences across mammalian taxa supports our hypothesis that these are functional regulatory sites.

In addition to PREs, multiple, high confidence miR binding sites were also predicted using the online prediction tool TargetScanHuman (v8.0) [72]. MiRs contain a critical binding sequence at nt positions 2–7 known as the seed sequence. Predicting miR binding motifs involves searching for sequences with perfect complementarity to the seed. Three types of sites are considered canonical: 8mers, 7mer-m8, and 7mer-A1 [73]. Sites of interest were narrowed using the following criteria: sites must match miR families broadly conserved among vertebrates or match the seed at positions 2–8 (8mer or 7mer-m8) in miR families poorly conserved among vertebrates, conserved/poorly conserved among mammals, or poorly conserved but confidently annotated. Of the numerous miR binding sites predicted by TargetScanHuman, 51 sites in *LRRK2*, 15 sites in *SNCA* and 5 sites in *SAT1* meet these criteria (Fig 1).

### Knockdown of PUM proteins in SH-SY5Y cells increases *LRRK2* and *SNCA* levels, but not *SAT1*

The presence of a PRE is a strong predictor of an mRNA being a PUM protein binding target. In a study conducted in *S*. *cerevisiae*, 42% of genes computationally predicted to contain a Puf3p binding motif in the 3’UTR were determined to be Puf3p targets through affinity isolation [38]. In humans, 69% of PUM1 and 74% of PUM2 binding targets contain at least one PRE in the 3’UTR [34]. It is less clear how many PUM binding targets are actually being regulated at the level of mRNA decay and/or translation. Based on the presence of perfect PREs in the 3’UTRs of *LRRK2* and *SNCA*, we hypothesized that these PD-associated genes are being regulated by one or both PUM proteins. Since PUM regulation is generally repressive, we expected alleviating PUM regulation would result in an increase in the expression levels of *LRRK2* and *SNCA*. Since the mutation in *SAT1* that is clinically relevant falls within a non-canonical PRE, we also tested regulation of *SAT1* by PUMs.

**Fig 2:**
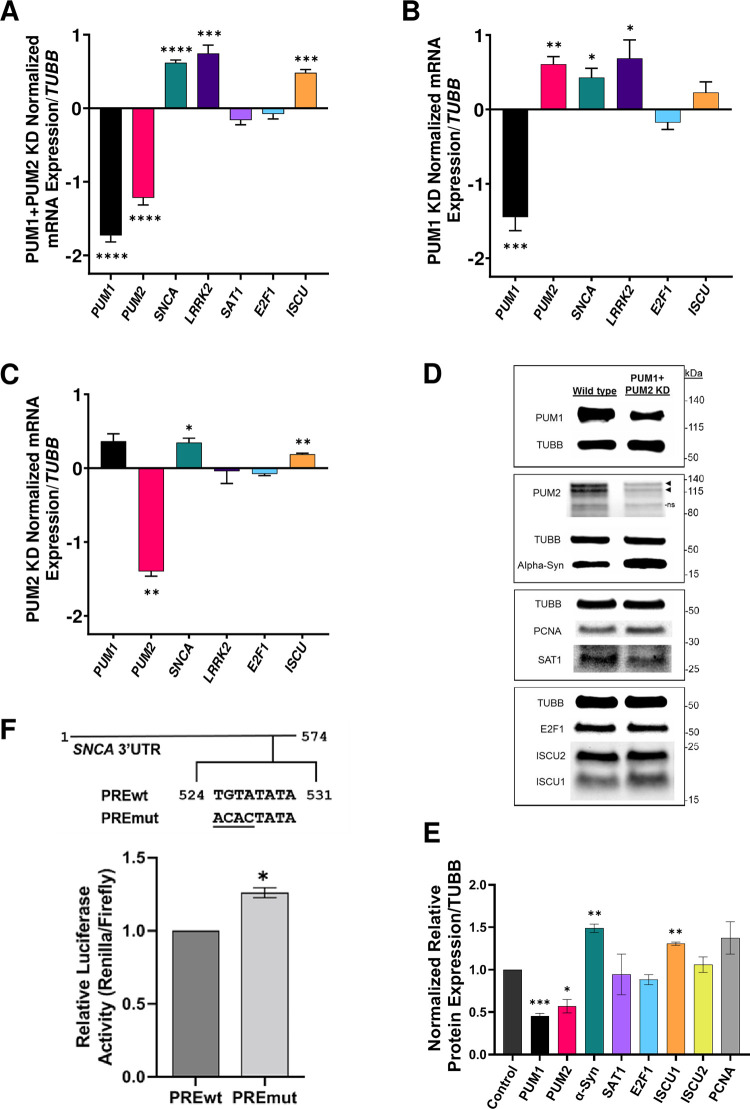
*LRRK2* and *SNCA* RNA and alpha-synuclein protein expression increase upon knockdown of human PUMs or PRE mutation. PUM1- and PUM2-targeting siRNAs were co-transfected into SH-SY5Y cells using electroporation to induce KD. Non-targeting siRNAs were also transfected into cells from the same population to serve as a negative control and establish relative WT levels of expression. RNA expression upon (A) PUM1 and PUM2 KD, (B) PUM1 only KD, and (C) PUM2 only KD was measured through RT-qPCR. All KDs were performed in technical triplicate and a minimum of biological triplicate. Each bar represents an analyzed mRNA. *E2F1* and *ISCU* served as negative and positive controls, respectively, for PUM regulation. RT-qPCR data was normalized to Beta Tubulin (*TUBB*) expression. Fold change of normalized mRNA levels in KD condition versus WT was calculated using 2^-ΔΔCT^, with values Log2 transformed and plotted on a linear y-axis. (D) Protein levels were assessed through Western blotting following double PUM1 and PUM2 KD. Shown are representative band patterns for each target from a single biological replicate. Proteins probed on the same blot are outlined in rectangles. The molecular weight markers (kDa) on the right represent the locations of protein ladder bands from each blot. Raw blot images can be viewed in full in S1 Fig. Probing for PUM2 resulted in the appearance of a non-specific band (ns) in addition to the expected bands noted by the black arrows. (E) Protein bands were quantified using BioRad Image Lab software and normalized to Beta Tubulin. E2F1 served as a negative control. ISCU and PCNA were used as positive controls for PUM regulation. The two isoforms of ISCU were quantified separately. Proteins were tested in a minimum of biological triplicate. (F) Relative normalized luciferase activity of reporters containing WT (PREwt) or mutated (PREmut) *SNCA* 3’UTRs. The location and sequence of the PRE and mutated nts on the *SNCA* 3’UTR is illustrated above the graph. All expression results were statistically analyzed using a paired, 2-tailed T-test. * = p-value ≤0.05, ** = p-value ≤0.01, *** = p-value ≤0.001, and **** = p-value ≤0.0001.

Human PUM1 and PUM2 are very similar in sequence, sharing 83% overall similarity and 97% similarity within the critical PUM homology domain, as well as target overlap [74]. Mammalian PUMs bind to thousands of mRNA targets and regulate a broad range of targets. Attempts to quantify human *PUM* targets using various techniques (RIP-Chip, PAR-CLIP, Bric-seq, and RNA-seq) have produced varying results, ranging from 700 to 3300 transcript targets with 82% to nearly 90% target redundancy between the two PUM proteins [34,37,48,75]. Due to the large degree of sequence and functional redundancy that exists between PUM1 and PUM2, we expected one of the PUMs would at least partially compensate for the absence of the other in a single PUM KD. Mammalian PUMs have also been found to regulate themselves and one another; knocking out one PUM results in a compensatory upregulation in the other PUM, often impeding assessment of genuine target regulation in a single PUM-depleted background [76,77]. Therefore, we first assessed the effect of knocking down both PUMs simultaneously in SH-SY5Y cells (Fig 2A). Through transfection of PUM-targeting siRNAs, we achieved a nearly 70% and 60% reduction in *PUM1* and *PUM2* mRNA, respectively. The reduction in both PUMs resulted in a significant increase in the expression of *SNCA* and *LRRK2*, but not in *SAT1*. *ISCU* and *E2F1* were used as controls for PUM regulation. *ISCU* is a confirmed PUM target and serves as a positive control [48]. *E2F1* serves as a negative control as it does not contain a PRE and it has been demonstrated that a KD of PUMs does not affect E2F1 protein expression [78]. Thus, PUM proteins are involved in the regulation of the proper levels of *SNCA* and *LRRK2* in SH-SY5Y cells.

To analyze the regulatory contribution of each PUM protein individually on the mRNA targets, we performed single KDs of each PUM (Fig 2B and 2C). Knockdown of PUM1 caused a more than 50% increase in *PUM2* mRNA levels, similar to previous studies [76]. Yet, the resulting pattern of mRNA expression change for *SNCA*, *LRRK2*, and the controls was similar to the double KD, despite the change in *ISCU* not reaching statistical significance (Fig 2B). Interestingly, KD of PUM2 did not induce the same magnitude of change in *PUM1* as the KD of PUM1 did in *PUM2*. The resulting increase in *SNCA* and *ISCU* was slightly lower than in the PUM1 KD (Fig 2C), indicating that these targets are regulated by both PUM proteins, but the strength of regulation may be unequal. Interestingly, *LRRK2* levels did not increase in the PUM2 KD, suggesting that this target is more affected by changes in PUM1 than PUM2. In fact, the increase in PUM1 levels in the PUM2 KD may be counter-acting any effect of reduced PUM2 levels. Since changes were not seen at the *SAT1* transcript level in the double KD, we did not test *SAT1* levels in single KDs.

Protein levels were also assessed after double KD of PUMs (Fig 2D and 2E). An approximately 50% reduction in both PUMs resulted in a 51% increase in α-syn. LRRK2 protein bands could not be detected from SH-SY5Y cell lysates. LRRK2 is very lowly expressed in SH-SY5Y cells, making visualization and quantification of endogenous protein difficult [12]. According to information on Human Protein Atlas (proteinatlas.org), average *LRRK2* mRNA expression in SH-SY5Y cells is 0.3 transcripts per million [79]. SAT1 protein levels were not significantly different between WT and PUM deficient conditions. The negative and positive controls used in qPCR testing, *E2F1* and *ISCU* respectively, were also tested by Western. As expected, E2F1 protein levels did not change significantly. Probing for ISCU produced two distinct bands. Alternative splicing of *ISCU* mRNA produces two protein isoforms. One isoform, ISCU1, is shorter (~15kDa) and localizes to the cytosol. ISCU2 is longer (~18kDa) and localizes to the mitochondria [80]. The bands were quantified separately, and only cytosolic ISCU1 levels were significantly affected by PUM KD. *PCNA* is also a confirmed target of PUM1 and serves as another positive control for PUM regulation [48]. PCNA protein levels increased almost 40% on average in the PUM KD cells; however, variability among biological replicates prevented statistical significance. The increase in expression of *SNCA* at the RNA and protein levels and *LRRK2* at the RNA level in the PUM-depleted cellular background demonstrates that these genes are regulated by PUM proteins.

To test direct regulation of *SNCA* by PUM proteins, we generated reporters that contain the WT or mutated 3’UTR of *SNCA* downstream of a *Renilla* luciferase gene. The mutated reporter contains an ACAC in place of TGTA at positions 1–4 of the *SNCA* canonical PRE (Fig 2F). Such a mutation has been shown to disrupt PUM binding [23,27,35,81,82]. In the WT PUM background of SH-SY5Y cells, luciferase activity of the reporter harboring the PRE mutation increased by 26% compared to the reporter with the WT PRE (Fig 2F). This demonstrates that the increase in *SNCA* expression observed in PUM-deficient conditions can be attributed, at least in part, to a direct interaction between PUM proteins and the *SNCA* 3’UTR at the canonical PRE.

### Small RNA-seq determination of global and differential miR expression in SH-SY5Y cells

**Fig 3:**
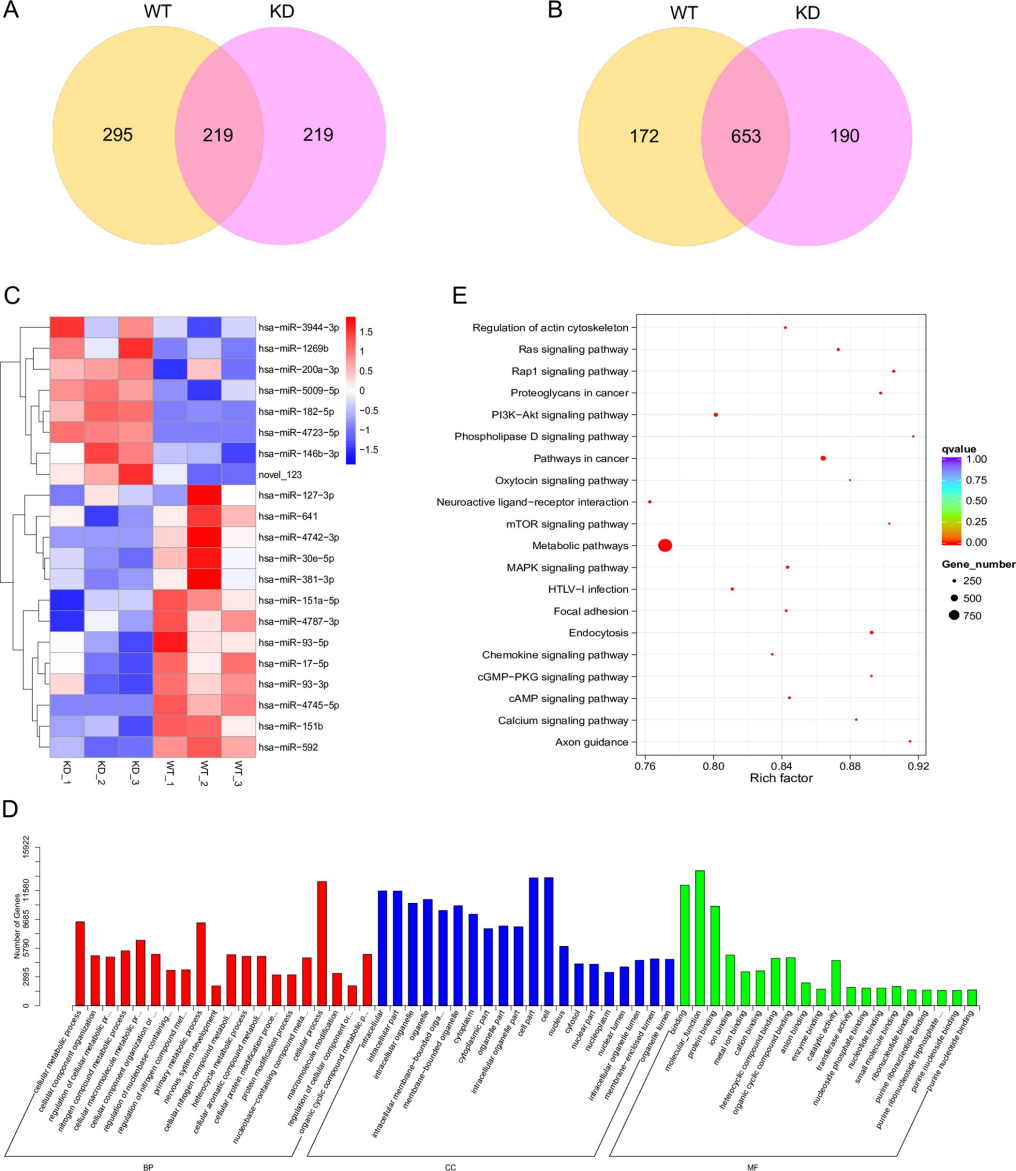
MicroRNA differential expression and pathway enrichment of predicted MicroRNA targets. (A) Venn diagram of the number of unique microRNAs determined to be expressed in either WT PUM background (purple) or KD PUM background (yellow), or co-expressed in both treatments (pink; overlap) based on raw sequence reads. (B) Venn diagram defining the number of microRNAs in WT PUM only (purple), PUM KD only (yellow), or both treatments (pink; overlap) that were determined to be suitable for differential expression analysis. (C) Heatmap of DEMs hierarchically clustered. Changes in log2(ratio) expression are represented as a color change from red to blue or vice versa. (D) Enrichment of GO terms among the predicted targets of DEMs. (E) Analysis of KEGG pathway enrichment for the top 20 pathways associated with targets of DEMs within the areas of Biological Process (BP), Cellular Component (CC), and Molecular Function (MF).

In addition to PUM regulation of the PD-associated genes, each of the studied genes has multiple predicted miR binding sites in their 3’UTRs. To evaluate the potential effects of miR regulation on expression of these PD-associated genes, we first wished to determine which of the miRs predicted to target *LRRK2* and *SNCA* are expressed in the SH-SY5Y cell line. Since PUM regulation and miR regulation of mRNA targets have been proposed to be linked through varying mechanisms, we also wanted to determine if PUM KD causes differential expression of any miRs. To elucidate the full miR expression profile of SH-SY5Y cells, we performed sRNA-seq in triplicate on WT SH-SY5Y cells and SH-SY5Y cells that were knocked down for both PUM1 and PUM2. Bowtie was used to map small RNA reads to the human genome [63]. Mapped sequences were compared to known mature and precursor miR sequences using miRbase20.0 as reference. MiREvo and mirdeep2 were employed to predict novel miRs from miR precursor hairpin structures [64,65]. Raw reads were discovered for 1404 total individual known and novel miRs between all samples, with 1185 present in WT samples and 890 present in both treatments (Fig 3A, S3 Table). Raw read counts were normalized to Transcripts Per Million (TPM) [66].

While PUMs can directly regulate target mRNAs, an additional layer of complexity could involve PUM regulation of miRs. Therefore, differential miR expression between WT and PUM KD samples was determined using DESeq2 [69]. After the filtering steps of the differential expression protocol, 1015 of the miRs were determined to be suitable for downstream analysis. Of these, 653 miRs were co-expressed between WT and KD cells, with 172 detected only in WT and 190 only in KD (Fig 3B; S4 Table). We next cross-referenced the miRs predicted to bind *LRRK2* and *SNCA* with those determined to be expressed based on normalized read counts and those that passed the filtering process of differential expression analysis (S5 Table). This allowed us to verify that all miRs depicted in Fig 1 that were determined to be expressed based on TPM were fully evaluated for differential expression. Twenty-six of the 51 miRs predicted to target the *LRRK2* 3’UTR, 4 out of 15 miRs predicted to target the *SNCA* 3’UTR, and 3 out of 5 miRs predicted to target *SAT1* were determined to be expressed. Expressed miRs are bolded and miRs determined to be differentially expressed are denoted by an asterisk (*) on Fig 1.

Pros and cons exist for all miR target prediction resources. TargetScan and miRanda algorithms use different prediction parameters and differ in their sensitivity and false discovery rates [83]. We initially used TargetScanHuman 8.0 to search for miR sites in our genes of interest because it is more conservative; however, it is not as sensitive as miRanda [83]. When target genes for the expressed known and novel miRs were predicted using miRanda, the list of candidates was much longer than the list produced by TargetScan (S6 Table). Results were sorted according to Ensembl (v103.38) gene annotations to compile a list of miRs specifically predicted to target *LRRK2* (ENSG00000188906), *SNCA* (ENSG00000145335), *SAT1* (ENSG00000130066), *PUM1* (ENSG00000134644), and *PUM2* (ENSG00000055917). Based on miRanda, 448 miRs are predicted to target *LRRK2*, 187 are predicted to target *SNCA*, and 47 are predicted to target *SAT1*. All miRs predicted by TargetScan and determined to be expressed, those bolded on Fig 1, are also predicted by miRanda to target *LRRK2*, *SNCA*, and *SAT1* with the exceptions of miR-140-3p and miR-374b-5p in *SNCA*, and miR-186-5p in *SAT1*.

We also analyzed the miRs that are predicted by miRanda to target the *PUMs* and found *PUM1* has 233 and *PUM2* has 246 potential targeting miRs. The *PUMs* only share 44 targeting miRs in common, 39 known and 5 novel (S6 Table). Interestingly, the *PUM*s share more targeting miRs in common with *LRRK2* and *SNCA* than with each other. Since *SAT1* levels did not respond to PUM KD and has very few predicted miR sites, we did not make comparisons between *SAT1* and *PUM*s. *PUM1* shares 80 predicted targeting miRs with *LRRK2* and 20 with *SNCA*, 10 of which target both PD genes. *PUM2* shares 93 with *LRRK2* and 37 with *SNCA*, 13 of which target both PD genes. *PUM1* and *LRRK2* share five miRs that are both confidently expressed in SH-SY5Y and have high confidence binding sites on *LRRK2*: miR-205-5p, miR-181a-5p, miR-181b-5p, miR-181d-5p, and miR-186-5p. Since it is well established that *PUMs* and miRs are both typically repressive in their regulation, we speculated that the relationship between *PUMs* and miRs regulating the same target would be complementary. Potential regulation of *PUM1* and *LRRK2* by the same miR suggests the relationship may be more complex and multifaceted.

**Fig 4:**
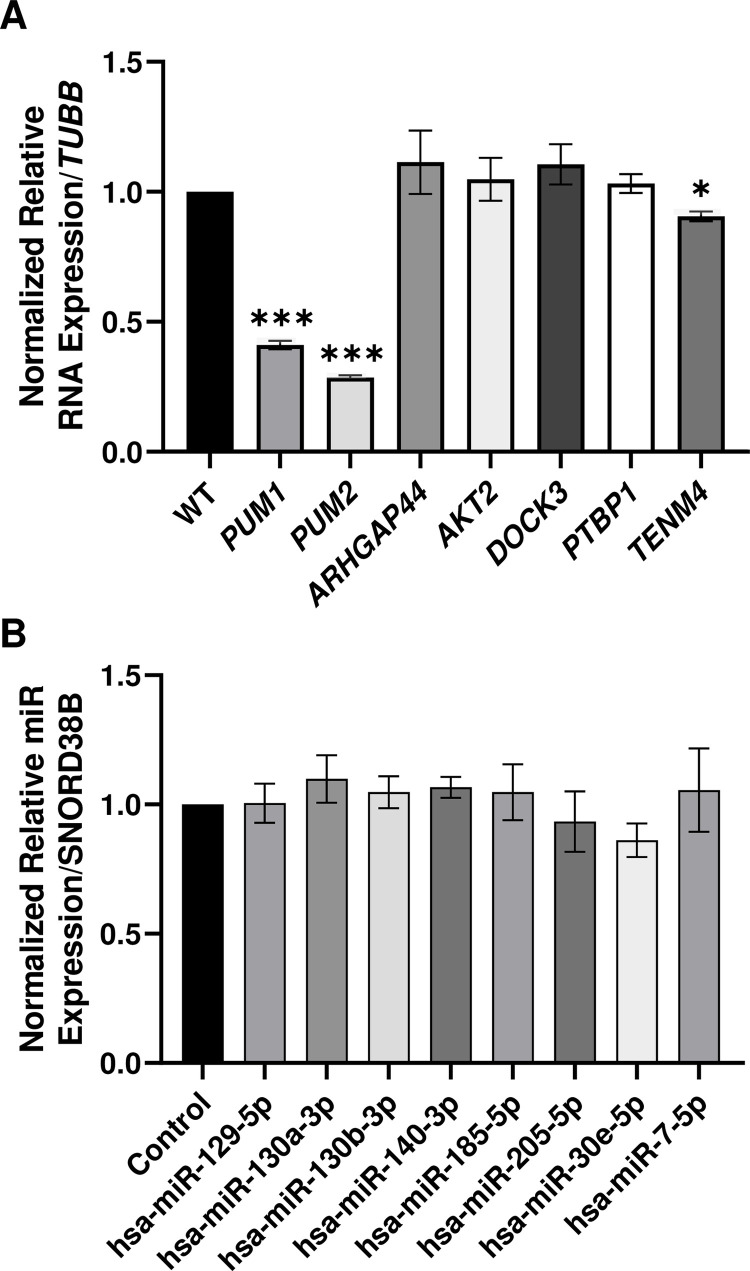
Relative expression of microRNAs and microRNA host genes in SH-SY5Y cells in wild type and PUM knockdown states. (A) Expression of host genes of miRs found in Table 1 that contain canonical PRE motifs in their 3’UTRs was measured using RT-qPCR. (B) MicroRNAs determined to be expressed through sRNA-seq were quantified through RT-qPCR. The control bar represents the expression in WT cells. The remaining bars represent the relative expression of the indicated genes or microRNAs in PUM1/2 KD cells. RT-qPCR data was normalized to *TUBB* for RNA expression and *SNORD38B* for miR expression. Statistical significance was determined using paired, two-tailed Student’s T-tests. * = p-value ≤0.05, ** = p-value ≤0.01, *** = p-value ≤0.001, and **** = p-value ≤0.0001.

The DEseq2 analysis revealed 21 miRs determined to be significantly differentially expressed between WT and PUM KD samples (S4 Table). The Log2 (TPM ratio) between biological replicates from WT and KD samples are represented in a heatmap with hierarchical clustering of miRs according to similarity of expression change pattern (Fig 3C). The top 8 clustered miRs are upregulated and the lower 13 miRs are downregulated in PUM-deficient SH-SY5Y cells. To examine whether changes in expression may be associated with PUM regulation of miR host genes, we searched for PREs in the 3’UTRs of host genes for the DEMs that originate from coding regions of the genome. Results are outlined in Table 1. Sixteen of the miRs are expressed from the transcripts of protein coding genes and of those, five have one or more perfect PRE motifs in their 3’UTRs. Thirteen of those 16 have one or more non-canonical PREs. As a control, six random, non-DEMs were selected for PRE analysis. Two of the six host genes contained canonical PREs and five of the six contained non-canonical PREs, suggesting that direct PUM regulation of host genes may not be the primary factor in differential expression of miRs upon PUM KD. To test this idea, we knocked down PUM1 and PUM2 levels in SH-SY5Y and performed RT-qPCR to quantify RNA levels of host genes of four DEMs (1 upregulated and 3 downregulated) and 1 unregulated miR containing at least one canonical PRE in the 3’UTR (Fig 4A). None of the host genes of DEMs were statistically differentially expressed in the PUM-deficient state; however, *TENM4*, the host gene of one of the miRs not found to be differentially expressed, did exhibit a statistically significant change in RNA levels upon PUM KD. We expected PUM KD would result in increased expression if PREs were functional; however, *TENM4* RNA levels decreased. Our results suggest that differential expression of miRs is not a product of regulation of miR host genes by PUM proteins.

**Table 1:** PREs in host genes of differentially expressed miRs.

	DEseq2 Results					Manual search of 3’UTRs			
	miRNA (hsa-miR-)	KD_read count	WT_read count	log2 Fold Change	pval	Host Gene	NCBI RefSeq	# cPRE	# ncPREs
UPREGULATED	5009-5p	15.423	1.928	1.401	0.0298	AP3S2	NM_005829.5	0	1
	3944-3p	18.695	2.704	1.269	0.0489	ECHS1	NM_004092.4	0	0
	**200a-3p**	**133.627**	**40.315**	**1.247**	**0.0313**	**unknown**	**N/A**	**N/A**	**N/A**
	4723-5p	6.596	0.000	1.209	0.0421	TMEM199	NM_152464.3	0	1
	novel_123	902.111	439.949	0.991	0.0001	lncRNA	XR_934960.2	0	4
	182-5p	206.508	100.765	0.973	0.0013	unknown	N/A	N/A	N/A
	146b-3p	165.948	93.492	0.760	0.0284	unknown	N/A	N/A	N/A
	1269b	4150.924	3155.166	0.389	0.0199	ARHGAP44*	NM_014859.6	1	3
DOWNREGULATED	93-5p	6222.189	8370.860	-0.418	0.0320	MCM7	NM_005916.5	0	1
	17-5p	261.926	392.089	-0.559	0.0303	lncRNA	NR_027350.1	2	19
	151a-5p	177.550	278.509	-0.616	0.0409	PTK2	NM_001352694.2	0	1
	**30e-5p**	**277.386**	**456.769**	**-0.672**	**0.0327**	**NFYC**	**NM_001142588.2**	**0**	**0**
	592	66.639	123.369	-0.818	0.0171	GRM8	NM_000845.3	0	2
	641	26.997	72.951	-1.116	0.0343	AKT2*	NM_001626.6	1	2
	93-3p	22.742	63.801	-1.137	0.0359	MCM7	NM_005916.5	0	1
	4742-3p	0.000	8.757	-1.199	0.0437	WDR26	NM_025160.7	1	12
	4787-3p	5.691	25.635	-1.298	0.0401	DOCK3*	NM_004947.5	3	2
	151b	5.847	24.139	-1.342	0.0282	EVL	NM_016337.3	0	0
	4745-5p	0.000	8.094	-1.363	0.0257	PTBP1*	NM_002819.5	1	3
	127-3p	52.116	235.150	-1.396	0.0235	RTL1	NM_001134888.3	0	0
	381-3p	53.302	197.874	-1.495	0.0042	lncRNA	NR_104192.1	0	0
UNREG	125a-5p	3072.247	3221.872	-0.067	0.7446	SPACA6	NM_001316972.2	0	0
	27b-3p	10073.576	11623.339	-0.203	0.1908	AOPEP	NM_001193329.3	0	1
	3184-5p	62009.295	49324.221	0.321	0.1347	NSRP1	NM_001261467.2	0	3
	4473	436.469	340.640	0.344	0.2021	MLLT3	NM_004529.4	1	14
	532-5p	5764.185	5526.571	0.059	0.7460	CLCN5	NM_000084.5	0	12
	708-3p	2004.533	1845.398	0.116	0.5855	TENM4*	NM_001098816.3	3	5

Table 1: This table outlines DEseq2 analysis results and host gene assessments for 21 DEMs and six randomly chosen non-DEMs. WT_readcount and KD_readcount columns represent the average normalized number of reads for miRs in either WT or PUM1/PUM2 KD conditions in SH-SY5Y cells across three biological replicates. The change in expression is listed as Log2 fold change and direction of change is indicated by positive or negative numbers. The p-value represents the significance value corrected using the Benjamini and Hochberg method. A p-value of 0.05 was used to determine significant differential expression. If miRs are encoded within protein-coding genes, the gene name is listed in the Host Gene column. Three miRs are encoded within long non-coding RNAs (lncRNAs) and three miRs are encoded in regions of the genome with no annotation (unknown). Each host gene or lncRNA is associated with a transcript listed in the NCBI RefSeq column. The 3’UTRs of each transcript were searched for the presence of canonical (cPRE) and non-canonical (ncPRE) PREs and the number of discovered motifs was recorded in the table. The row for DEMs predicted to target *SNCA*, *LRRK2*, or *SAT1* are bolded. Host genes that were tested for changes in RNA expression in WT versus PUM KD states (Fig 4A) are indicated by an asterisk (*) in the Host Gene column.

Five of the 21 DEMs are predicted to target PD-associated genes of interest. Only two DEMs are represented on Fig 1, with miR-30e having one and miR-200a-3p having two putative binding sites in the *LRRK2* 3’UTR. The other DEM predicted to bind *LRRK2* is miR-381-3p, which has one site but is not represented on the 3’UTR map because it did not meet our criteria for inclusion, only matching position 2–7 of the seed sequence. The full length 3’UTR of *SNCA* has four predicted DEM sites: miR-182-5p, miR-381-3p, miR-93-5p, and miR-17-5p. Alignment of *SNCA* expressed sequence tags revealed four possible polyadenylation events, with 95% of all *SNCA* transcripts having a 3’UTR ≤574 nts [22]. We are using the 574 nt 3’UTR length for experimentation, so the 3’UTR map of *SNCA* is limited to this length. MiR-381-3p has a single site within the first 574nts, but the site only matches nts 2–7 of the seed sequence, so it did not meet our criteria for being added to the map. MiR-182-5p has a single site, and miR-93-5p and miR-17-5p both have two sites; however, all of these predicted sites are located downstream of nt 574, meaning they only potentially affect 5% of *SNCA* transcripts. MiR-182-5p and miR-200a-3p are upregulated, while the other three *LRRK2* and *SNCA*-related DEMs are downregulated in PUM-deficient cells, again suggesting that the relationship between PUM and miR regulation is complex.

To better understand the potential effects of the DEMs on cellular biological functions, we looked for enrichment in Gene Ontology and KEGG pathway terms amongst the predicted gene targets of DEMs, paying particular attention to enrichments in neuronal and PD-related terms. DEM target gene candidates were mapped to GO terms in the database at www.geneontology.org and enrichment was calculated based on number of target genes mapped to a GO term relative to the reference gene background of that term (S7 Table). Fig 3D demonstrates the top 20 most enriched GO terms in each of the three GO categories: Biological Process (BP), Cellular Component (CC), and Molecular Function (MF). The top 10 GO terms for each category were also organized into Directed Acyclic Graphs (DAGs) to visualize the relationships between enriched terms (S5 Fig). Based on the GO enrichment and DAGs, differential expression of miRs brought about by downregulation of PUM proteins is predicted to impact cell metabolic pathways and component organization in the intracellular space, both cytosolic and membrane-bound organelle associated.

Performing a KEGG pathway enrichment analysis allowed us to identify the major pathways in which the DEM target gene candidates are involved. The top 20 most enriched KEGG pathways are ranked according to their statistical enrichment (S8 Table) and are demonstrated in Fig 3E. Richness within a pathway is measured as a ratio of the number of DEM target genes associated with a pathway to the total number of genes associated with that pathway. More than half of the top pathways are signaling pathways. The term with the lowest q-value is Metabolic Pathways. Nearly 1000 target gene candidates are linked to the Metabolic Pathways term, but this is not surprising considering the prominence of metabolic pathways in the BP GO terms. Two neuron specific pathways, neuroactive ligand-receptor interaction and axon guidance, rank in the top 20. We also see two of the top 20 most enriched pathways associated with cancer. Since PD is our disease of interest in this study, we searched for Parkinson’s Disease in the KEGG terms. We discovered that out of 142 reference genes that are associated with PD, 80 are target gene candidates of the DEMs, giving this term a richness factor of 0.56 and a q-value of 0.00345. These results suggest that disruptions in the DEMs collectively may have an impact on important metabolic and intracellular signaling processes, as well as on diseases including PD. **Expression of miR sequences in SH-SY5Y cells**

To further analyze miRs found to be expressed in SH-SY5Y cells, we chose 10 candidate miRs with predicted binding sites spread out along the length of *SNCA* and *LRRK2* 3’UTRs, with special consideration given to sites close to putative PREs. Our goal was to ascertain the relative levels of mature miRs in the cells by RT-qPCR and whether the differential expression of miR-200a and miR-30e seen in sRNA-seq results could be confirmed empirically. Fig 4B depicts the relative normalized expression of mature miRs of interest between WT and PUM-deficient SH-SY5Y cells, using *SNORD38B* as our normalization control. *SNORD38B* displayed the most stable expression of all the reference gene candidates that were assessed (S2 Fig). Our results corroborated sRNA-seq findings that the following miRs are not differentially expressed in SH-SY5Y: hsa-miR-129-5p, hsa-miR-130a-3p, hsa-miR-130b-3p, hsa-miR-140-3p, hsa-miR-185-5p, hsa-miR-205-5p, hsa-miR-323a-3p, and hsa-miR-7-5p (Fig 4B). Equivocating average CT values to relative expression among miRs, hsa-miR-205-5p and hsa-miR-200a-3p were very lowly expressed. Using the maximum recommended amount of input RNA, which was four times higher than the amount used to quantify all other miRs, and a dilution factor of only 1:6, hsa-miR-205-5p had a CT > 35 for both treatments and hsa-miR-200a-3p did not reach threshold by the end of cycle 39. For this reason, hsa-miR-200a-3p is not included in the graph and differential expression could not be confirmed nor refuted. Hsa-miR-30e-5p was determined to be downregulated in PUM KD cells by sRNA-seq analysis, and while a decrease in expression in PUM KD was observed, the change in expression did not reach statistical significance when measured by miR RT-qPCR.

## Discussion

Only 10–15% of PD cases can be attributed to heritable predisposition, with only 5–10% of those cases being traced to monogenic, autosomal sources [84]. This means that 9 out of every 10 cases of diagnosed PD are considered sporadic, signifying cause unknown. We have only scratched the surface of a complete understanding of PD etiology. The list of genes determined to be disease inducing is currently at 19 and the list of at-risk loci is even longer and constantly expanding [84]. In this study, we provide evidence that PUM proteins are another potential player in PD pathogenesis. Our results indicate PUMs regulate the expression of the clinically relevant PD genes *SNCA* and *LRRK2*, and that cellular alteration in PUM levels leads to increased mRNA expression of these PD-associated genes and a significant increase in the abundance of the α-syn protein. With overexpression and aggregation of α-syn one of the defining features of PD, the role of PUMs in regulating α-syn levels is of high importance. One microarray study reports that PUM1 and PUM2 levels decrease in the brain tissue of some PD patients, providing further support for a link between PD and PUMs [85]. Moreover, despite substantial target redundancy and co-regulation by PUMs [34,37,48,75–77], a decrease in either PUM homolog individually or in tandem resulted in an increase in *SNCA*, demonstrating the sensitivity of this gene to any change in cellular PUM levels. In contrast, *LRRK2* mRNA expression was more susceptible to changes in PUM1levels, indicating that regulation by PUM1 and PUM2 is not always redundant. It is likely that the assembly of different proteins and miRNAs on different 3’UTR sequences promotes PUM-specific effects.

PUM proteins are RBPs that regulate targets through direct interactions with PREs in the target transcript, typically in the 3’UTR. This mechanism of regulation presents a second opportunity for PUM-based dysregulation of *SNCA* or *LRRK2* besides changes in PUM levels in the cell; mutations that arise in functional PREs located in *SNCA* or *LRRK2* can prevent PUM binding, alleviating PUM regulation and leading to increased expression. Our studies demonstrate that mutation of the canonical PRE in the *SNCA* 3’UTR indeed results in increased *SNCA* expression, confirming the direct regulation by PUMs through this PRE. Further support for the functional significance of the PREs in *SNCA* and *LRRK2* is demonstrated through the conservation of these sites across mammalian taxa, especially for the canonical PREs and those located closest to the coding region. The NCBI dbSNP reports single nt polymorphisms in 10 different loci within the canonical PREs of the 3’UTRs of *SNCA* and *LRRK2*. Six of the SNP variants are located within the core UGUA. Clinical significance was not reported to the clinVAR database for any of the variants, suggesting that mutations in PREs can be found in the population, but no link to clinical PD has yet been established.

MicroRNAs are also big players in post-transcriptional regulation through 3’UTR recognition sites. While algorithms can predict miR target sites in PD-associated genes, not all miRs are expressed in a particular cell type. For studying PD, the SH-SY5Y human neuroblastoma cell line has been the standard line of choice, yet few studies have attempted to elucidate genome-wide miR expression in SH-SY5Y cells. Of these studies, only one utilized sRNA-seq [86], while the others used microarrays [87,88]. Microarrays only probe for known miRs, while sRNA-seq can detect novel miRs. It is also typical for different genome-wide studies to produce data sets that are not entirely overlapping, so a full picture of expressed miRs can only be seen with multiple studies. In our study, the intention was to both generate a comprehensive miR profile for SH-SY5Y cells and to assess the impact of PUM KD on miR expression. The use of sRNA-seq permitted us to detect 1154 miRs that mapped to known mature miRs as well as 250 novel miRs, one of which was differentially expressed upon PUM KD.

Comparing miR levels between WT and double PUM KD SH-SY5Y cells revealed 21 miRs differentially expressed in a PUM-deficient state. Most of the DEMs are produced from within protein coding genes (Table 1). In a search for PREs in the 3’UTRs of DEM host genes, we discovered that most of the host genes associated with DEMs had either perfectly conserved and/or nearly conserved PRE motifs. This would suggest PUMs may be involved to some degree in regulation of the DEM host gene expression. However, PREs were also identified in non-differentially expressed miRs. In testing four DEM host genes as well as a non-DEM host gene, we did not see detectable differences in expression upon PUM KD. Thus, PUM regulation of host genes is not a global mechanism for the miR expression differences seen in PUM KD cells. Of the 21 DEMS, only eight are upregulated. If PUM regulation of these transcripts is repressive, we would expect a decrease in PUM levels to result in increased expression of miRs produced from PUM-regulated genes. Studies elucidating PUM regulation in human cells have demonstrated that PUM deficiency can result in increased levels of some targets [37,48]. In 2018, Bohn et al. attempted to identify functional PUM targets. None of the host genes from Table 1 were found to be differentially expressed upon PUM KD in HEK293 cells, however, they confirm that three of the transcripts are physically bound by a PUM [37]. Given that PUMs are predicted to regulate thousands of genes, it is entirely possible that some differentially expressed genes upon PUM KD are secondary targets rather than primary targets of PUM regulation.

While the cause of the differential expression of this subset of miRs remains elusive, the DEMs further support our assertion that PUMs are involved in the regulation of PD through both direct regulation of target mRNAs and through indirect mechanisms involving miRs. Six DEMs have pre-existing associations with PD or neurodegeneration. MiR-200a-3p and miR-182-5p were found to be upregulated, while miR-127-3p was found to be downregulated, in the cerebrospinal fluid samples from PD patients compared to healthy patients [55,56]. The direction of expression change in these clinical samples matches the direction of change induced in miRs in a PUM-deficient SH-SY5Y cellular background. MPTP is a structural analog of dopamine that gets metabolized into a toxin that preferentially destroys dopaminergic neurons, thereby inducing parkinsonism through neuronal loss in the SN [89]. It is considered a classic model for the study of PD using mice. The cells of the SNpc of mice treated with MPTP have decreased miR-30e and miR-93 expression [90,91]. Mice administered a mir-30e agomir displayed increased levels of miR-30e as well as improved motor function and attenuated neuronal loss [90]. MiR-93, both arms of which are differentially expressed, appears to serve a neuroprotective role, downregulating *STAT3* to decrease inflammatory response and apoptosis in neurons [91]. As we reported in Fig 1, miR-200a-3p, miR-182-5p, miR30e-5p, and miR-93-5p are predicted to target *LRRK2* and *SNCA*.

We are using SH-SY5Y cells as a neuronal model to study the link between PUM regulation and PD, so to analyze the miRs involved in these pathways, we searched GO and KEGG pathway enrichments in DEMs related specifically to neurons and PD. We found that 1233 predicted DEM targets were related to the GO cellular component term “neuron,” and the GO biological processes of “neurogenesis, neuronal differentiation, neuron projection development, and neuron development” were all enriched with over 800 predicted targets mapping to each. Among the 279 KEGG pathways found to be significantly enriched for DEM targets, “Parkinson’s Disease” and “dopaminergic synapse” pathways were present. In the Parkinson’s disease pathway, 80 out of 142 genes related to this pathway were predicted targets of our DEMs. Therefore, in addition to direct regulation of PD-associated genes, PUM proteins may also impact PD pathways through regulation of miRs. Future studies will further elaborate the mechanistic roles of PUM proteins and miRs in the regulation of Parkinson’s disease.

## Supporting information

S1 Fig Annotated raw blots. (PDF)

S2 Fig Relative small RNA reference gene candidate expression across biological replicates in WT vs PUM KD SH-SY5Y. (PDF)

S3 Fig Predicted PRE locations in 3’UTR sequences of *LRRK2*, *SNCA*, and *SAT1*. (PDF)

S4 Fig Sequence conservation within putative canonical (cPRE) and non-canonical (ncPRE) PREs among mammals. (PDF)

S5 Fig Directed acyclic graphs for GO top 20. (PDF)

S1 Table Primers for RT-qPCR and site-directed mutagenesis. (XLSX)

S2 Table Antibodies for western blotting. (XLSX)

S3 Table Known and novel miR raw read counts. (XLSX)

S4 Table Differential expression analysis. (XLSX)

S5 Table MiRs predicted to be in the 3’UTR of *SAT1*, *LRRK2*, and/or *SNCA* based on TargetScanHuman db v8.0. (XLSX)

S6 Table Known mature and novel miRNAs predicted by miRanda to target *PUM1*, *PUM2*, *SNCA*, *LRRK2*, and *SAT1*. (XLSX)

S7 Table GO term enrichment for DEM targets. (XLSX)

S8 Table KEGG pathway enrichment analysis. (XLSX)
