# Supplementary material for: Regulation of Parkinson’s disease-associated genes by Pumilio proteins and microRNAs in SH-SY5Y neuronal cells
Source: PLoS One. 2022 Sep 29;17(9):e0275235. doi: 10.1371/journal.pone.0275235 (PMC9522289; doi:10.1371/journal.pone.0275235)
Supplement: S1 Fig — (PDF) [file pone.0275235.s001.pdf]

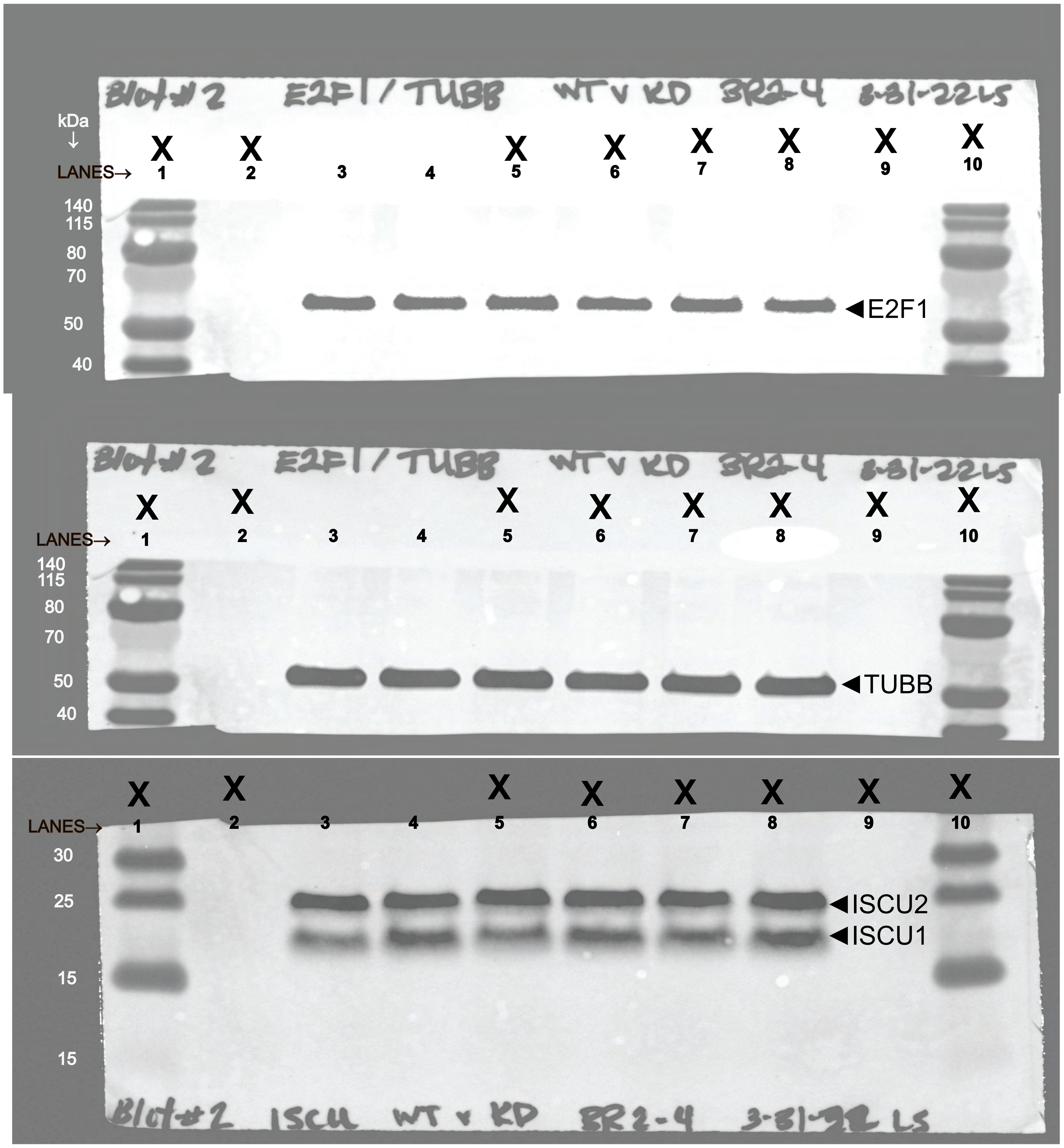

First Probe = E2F1

Second Probe = TUBB

WB images corresponding to Figure 2D:
Probing for E2F1, ISCU, and TUBB protein on nitrocellulose membrane after separation in 10% Bis-Tris Gel (MES-based buffer):

Lanes:
1. Ladder - PageRuler (Thermo Scientific 26616)
2. X (Loaded with RIPA and Load dye)
3. Bio Rep 1 - whole cell lysate of SH-SY5Y cells transfected with Negative control siRNA (WT expression)
4. Bio Rep 1 - whole cell lysate of SH-SY5Y cells transfected with PUM1 and PUM2 siRNAs (Expression in KD condition)
5. Bio Rep 2 - whole cell lysate of SH-SY5Y cells transfected with Negative control siRNA (WT expression)
6. Bio Rep 2 - whole cell lysate of SH-SY5Y cells transfected with PUM1 and PUM2 siRNAs (Expression in KD condition)
7. Bio Rep 3 - whole cell lysate of SH-SY5Y cells transfected with Negative control siRNA (WT expression)
8. Bio Rep 3 - whole cell lysate of SH-SY5Y cells transfected with PUM1 and PUM2 siRNAs (Expression in KD condition)
9. X (Loaded with RIPA and Load dye)
10. Ladder - PageRuler (Thermo Scientific 26616)

Post-Transfer membrane was cut between markers 30 kDa and 40 kDa
- Lower portion of blot probed with anti-ISCU antibody
- Upper portion of blot probed first with anti-E2F1 antibody. After imaging, blot was stripped and re-probed with anti-TUBB antibody

Chemiluminescent signal imaged and analyzed with BioRad ChemiDoc imager and Image Lab v6.1 software
High and Low intensities (but not Gamma) were adjusted across the entire image to improve band resolution. Adjustments were verified to NOT impact quantification of bands.
NS = non-specific bands

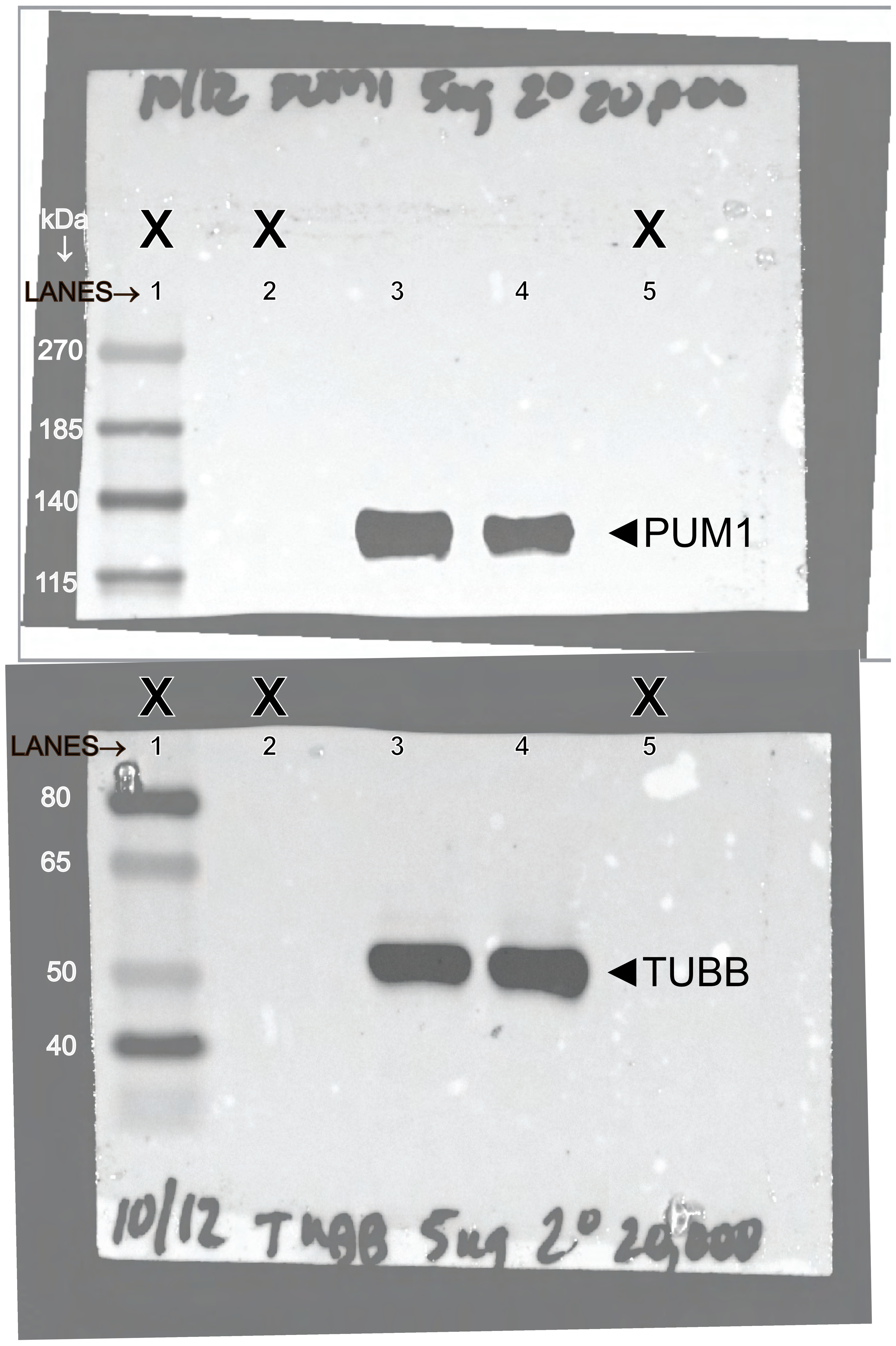

W**B images corresponding to Figure 2D:**
Probing for PUM1 and TUBB protein on nitrocellulose membrane after separation in 10% Bis-Tris Gel (MOPS-based buffer):

Lanes:
1. Ladder - Spectra Multicolor High Range Protein Ladder (Thermo Scientific 26625)
2. X (Loaded with RIPA and Load dye)
3. whole cell lysate of SH-SY5Y cells transfected with Negative control siRNA (WT expression)
4. whole cell lysate of SH-SY5Y cells transfected with PUM1 and PUM2 siRNAs (Expression in KD condition)
5. X (Loaded with RIPA and Load dye)

Post-Transfer membrane was cut between markers 65 kDa and 50 kDa
Upper portionof blot probed with anti-PUM1 antibody
Lower portion of blot probed with anti-TUBB antibody

Chemiluminescent signal imaged and analyzed with BioRad ChemiDoc imager and Image Lab v6.1 software

High and Low intensities (but not Gamma) were adjusted across the entire image to improve band resolution. Adjustments were verified to NOT impact quantification of bands.

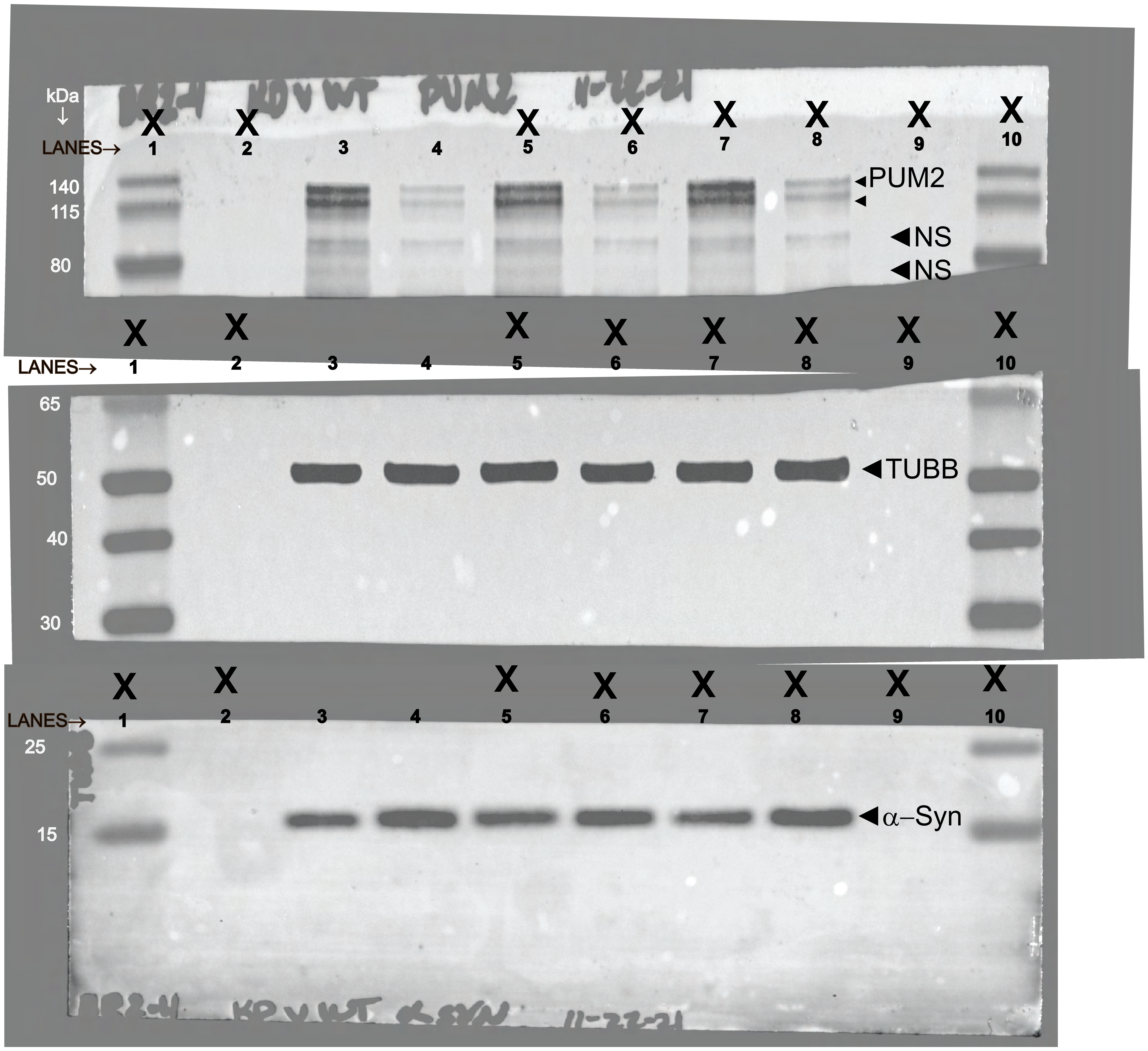

WB images corresponding to Figure 2D:
Probing for PUM2, alpha-synuclein, and TUBB protein on nitrocellulose membrane after separation in 10% Bis-Tris Gel:

Lanes:
1. Ladder - PageRuler (Thermo Scientific 26616)
2. X (Loaded with RIPA and Load dye)
3. Bio Rep 1 - whole cell lysate of SH-SY5Y cells transfected with Negative control siRNA (WT expression)
4. Bio Rep 1 - whole cell lysate of SH-SY5Y cells transfected with PUM1 and PUM2 siRNAs (Expression in KD condition)
5. Bio Rep 2 - whole cell lysate of SH-SY5Y cells transfected with Negative control siRNA (WT expression)
6. Bio Rep 2 - whole cell lysate of SH-SY5Y cells transfected with PUM1 and PUM2 siRNAs (Expression in KD condition)
7. Bio Rep 3 - whole cell lysate of SH-SY5Y cells transfected with Negative control siRNA (WT expression)
8. Bio Rep 3 - whole cell lysate of SH-SY5Y cells transfected with PUM1 and PUM2 siRNAs (Expression in KD condition)
9. X (Loaded with RIPA and Load dye)
10. Ladder - PageRuler (Thermo Scientific 26616)

Post-Transfer membrane was cut between markers 65 kDa and 80 kDa, as well as 25 kDa and 30 kDa
Upper portion of blot probed with anti-PUM2 antibody
Middle portion of blot probed with anti-TUBB antibody
Lower portion of blot probed with anti-Alpha Synuclein antibody

Chemiluminescent signal imaged and analyzed with BioRad ChemiDoc imager and Image Lab v6.1 software
High and Low intensities (but not Gamma) were adjusted across the entire image to improve band resolution. Adjustments were verified to NOT impact quantification of bands.
NS = non-specific bands

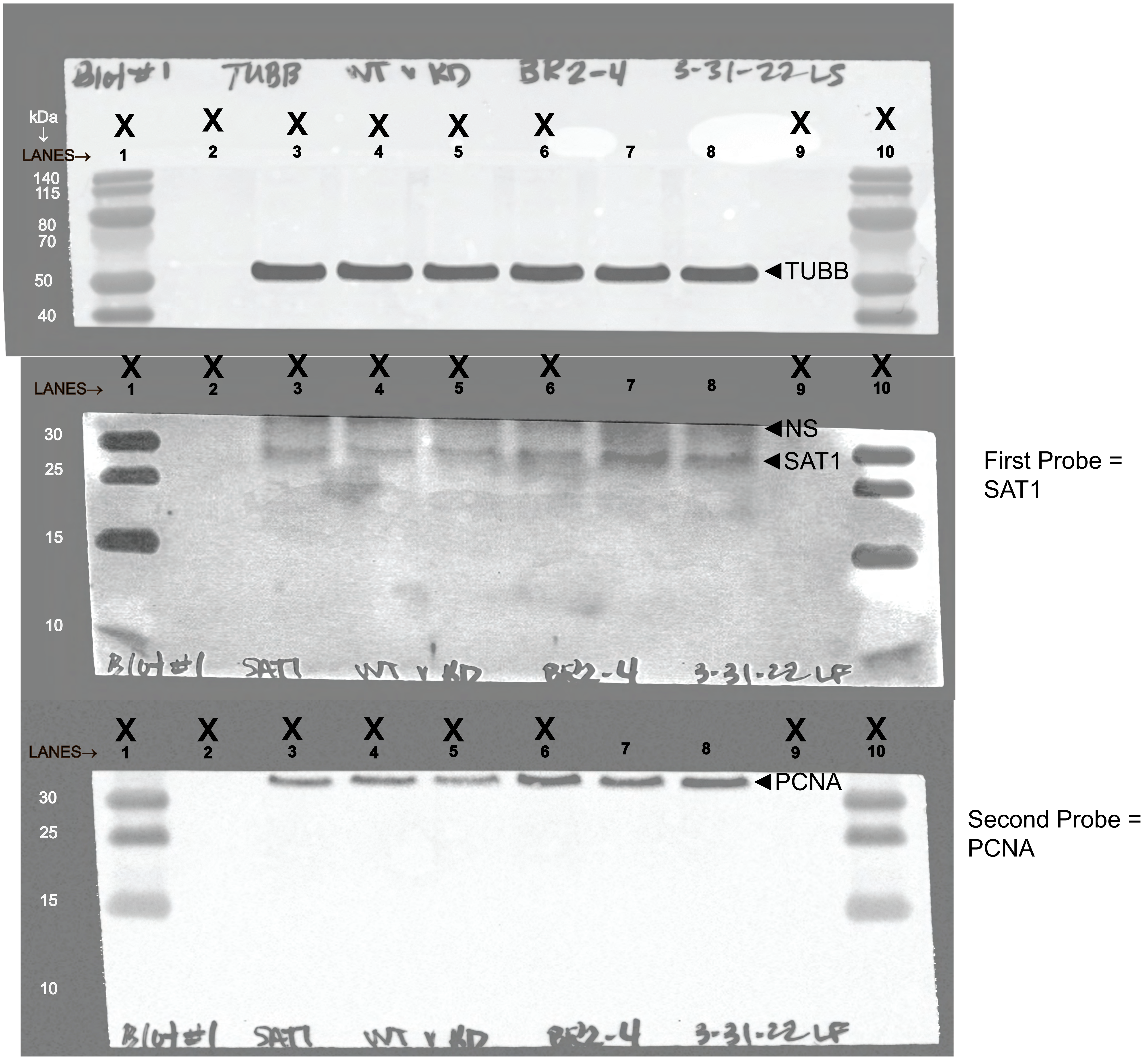

W**B images corresponding to Figure 2D:**

Probing for SAT1, PCNA, and TUBB protein on nitrocellulose membrane after separation in 10% Bis-Tris Gel (MES-based buffer):

Lanes:

1. Ladder - PageRuler (Thermo Scientific 26616)
2. X (Loaded with RIPA and Load dye)
3. Bio Rep 1 - whole cell lysate of SH-SY5Y cells transfected with Negative control siRNA (WT expression)
4. Bio Rep 1 - whole cell lysate of SH-SY5Y cells transfected with PUM1 and PUM2 siRNAs (Expression in KD condition)
5. Bio Rep 2 - whole cell lysate of SH-SY5Y cells transfected with Negative control siRNA (WT expression)
6. Bio Rep 2 - whole cell lysate of SH-SY5Y cells transfected with PUM1 and PUM2 siRNAs (Expression in KD condition)
7. Bio Rep 3 - whole cell lysate of SH-SY5Y cells transfected with Negative control siRNA (WT expression)
8. Bio Rep 3 - whole cell lysate of SH-SY5Y cells transfected with PUM1 and PUM2 siRNAs (Expression in KD condition)
9. X (Loaded with RIPA and Load dye)
10. Ladder - PageRuler (Thermo Scientific 26616)

Post-Transfer membrane was cut between markers 30 kDa and 40 kDa
Upper portion of blot probed with anti-TUBB antibody
Lower portion of blot probed first with anti-SAT1 antibody. After imaging, blot was stripped and re-probed with anti-PCNA antibody

Chemiluminescent signal imaged and analyzed with BioRad ChemiDoc imager and Image Lab v6.1 software
High and Low intensities (but not Gamma) were adjusted across the entire image to improve band resolution. Adjustments were verified to NOT impact quantification of bands.
NS = non-specific bands
