# Supplementary material for: Regulation of Parkinson’s disease-associated genes by Pumilio proteins and microRNAs in SH-SY5Y neuronal cells
Source: PLoS One. 2022 Sep 29;17(9):e0275235. doi: 10.1371/journal.pone.0275235 (PMC9522289; doi:10.1371/journal.pone.0275235)
Supplement: S2 Fig — (PDF) [file pone.0275235.s002.pdf]

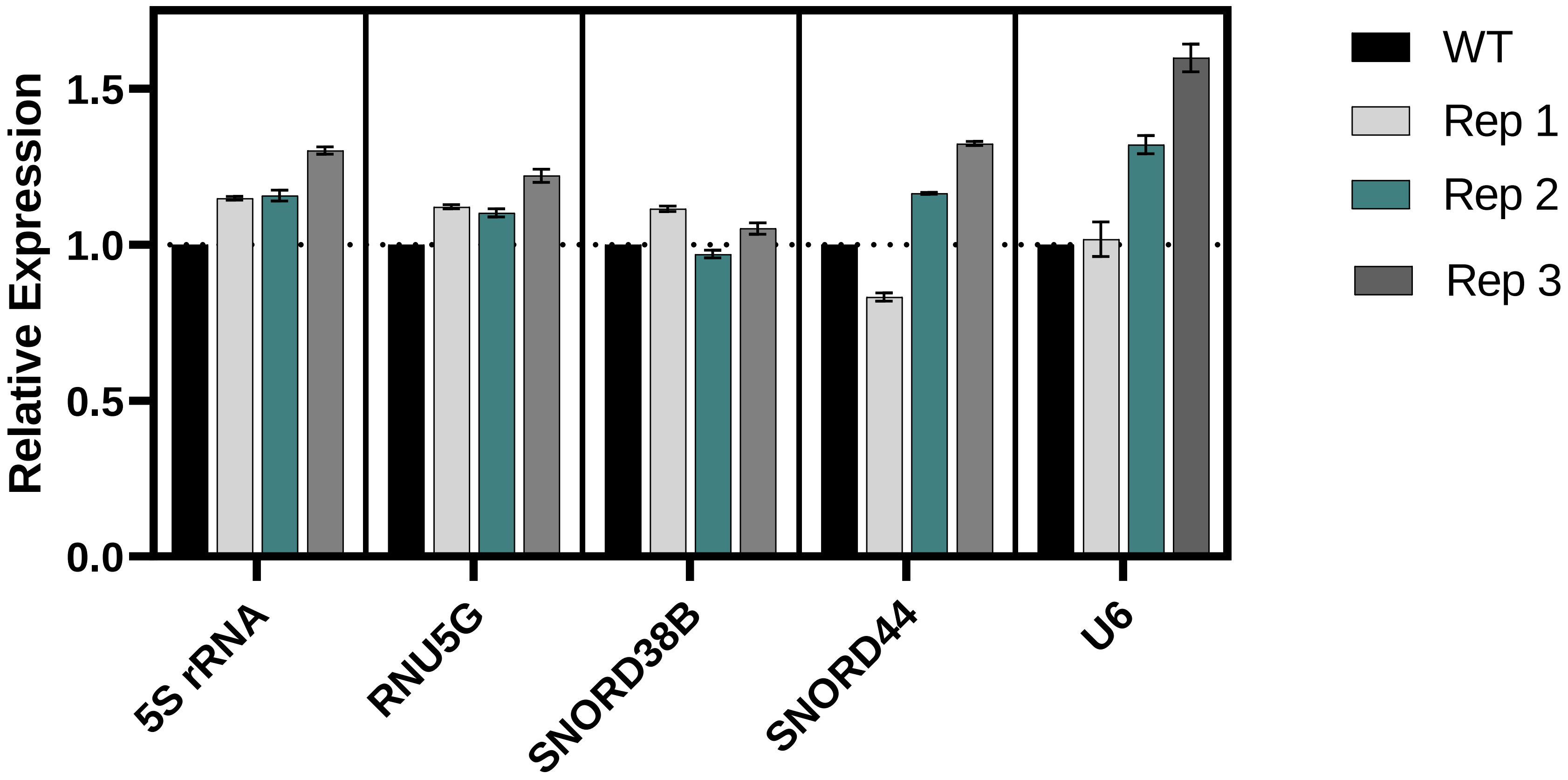

Figure S2. Relative small RNA reference gene candidate expression across biological replicates in WT vs PUM KD SH-SY5Y. Expression of five commonly used small RNA reference genes was assessed in SH-SY5Y cells treated with non-targeting (WT) or PUM1/2 siRNAs. Tests were performed in technical and biological triplicate. Expression is plotted on the y-axis as fold change ($2^{-\Delta\Delta CT}$) of each biological replicate in PUM KD cells relative to WT.
