# Supplementary material for: Regulation of Parkinson’s disease-associated genes by Pumilio proteins and microRNAs in SH-SY5Y neuronal cells
Source: PLoS One. 2022 Sep 29;17(9):e0275235. doi: 10.1371/journal.pone.0275235 (PMC9522289; doi:10.1371/journal.pone.0275235)
Supplement: S3 Fig — (PDF) [file pone.0275235.s003.pdf]

Figure S3. Predicted PRE locations in 3’UTR sequences of *LRRK2*, *SNCA*, and *SAT1*.

Canonical = TGTA+(A/T)+ATA
Non-canonical = TGTA + AT-rich
Not PRE = TGTA only

A) *LRRK2* 3’UTR

GAGAGAAATAGGAATTGTCTTTGGATAGGAAAATTATTCTCTCCTCTTGTAAATATTTATTTTAAAAATGTTCACAT
GGAAAGGGTACTCACATTTTTTGAAATAGCTCGTGTGTATGAAGGAATGTTATTATTTTTAATTTAAATATATGTAA
AAATACTTACCAGTAAATGTGTATTTTAAAGAACTATTTAAAACACAATGTTATATTTCTTATAAATACCAGTTACT
TTCGTTCATTAATTAATGAAAATAAATCTGTGAAGTACCTAATTTAAGTACTCATACTAAAATTTATAAGGCCGATA
ATTTTTTGTTTTCTTGTCTGTAATGGAGGTAAACTTTATTTTAAATTCTGTGCTTAAGACAGGACTATTGCTTGTCG
ATTTTTCTAGAAATCTGCACGGTATAATGAAAATATTAAGACAGTTTCCCATGTAATGTATTCCTTCTTAGATTGCA
TCGAAATGCACTATCATATATGCTTGTAAATATTCAAATGAATTTGCACTAATAAAGTCCTTTGTTGGTATGTGAAT
TCTCTTTGTTGCTGTTGCAAACAGTGCATCTTACACAACTTCACTCAATTCAAAAGAAAACTCCATTAAAAGTACTA
ATGAAAAAACATGACATACTGTCAAAGTCCTCATATCTAGGAAAGACACAGAAACTCTCTTTGTCACAGAAACTCTC
TGTGTCTTTCCTAGACATAATAGAGTTGTTTTTCAACTCTATGTTTGAATGTGGATACCCTGAATTTTGTATAATTA
GTGTAAATACAGTGTTCAGTCCTTCAAGTGATATTTTTATTTTTTTATTCATACCACTAGCTACTTGTTTTCTAATC
TGCTTCATTCTAATGCTTATATTCATCTTTTCCCTAAATTTGTGATGCTGCAGATCCTACATCATTCAGATAGAAAC
CTTTTTTTTTTTCAGAATTATAGAATTCCACAGCTCCTACCAAGACCATGAGGATAAATATCTAACACTTTTCAGTT
GCTGAAGGAGAAAGGAGCTTTAGTTATGATGGATAAAAATATCTGCCACCCTAGGCTTCCAAATTATACTTAAATTG
TTTACATAGCTTACCACAATAGGAGTATCAGGGCCAAATACCTATGTAATAATTTGAGGTCATTTCTGCTTTAGGAA
AAGTACTTTCGGTAAATTCTTTGGCCCTGACCAGTATTCATTATTTCAGATAATTCCCTGTGATAGGACAACTAGTA
CATTTAATATTCTCAGAACTTATGGCATTTTACTATGTGAAAACTTTAAATTTATTTATATTAAGGGTAATCAAATT
CTTAAAGATGAAAGATTTTCTGTATTTTAAAGGAAGCTATGCTTTAACTTGTTATGTAATTAACAAAAAAATCATAT
ATAATAGAGCTCTTTGTTCCAGTGTTATCTCTTTCATTGTTACTTTGTATTTGCAATTTTTTTTACCAAAGACAAAT
TAAAAAAATGAATACCATATTTAAATGGAATAATAAAGGTTTTTTAAAAACTTTAAA

B) *SNCA* 3’UTR

GAAATATCTTTGCTCCCAGTTTCTTGAGATCTGCTGACAGATGTTCCATCCTGTACAAGTGCTCAGTTCCAATGTGC
CCAGTCATGACATTTCTCAAAGTTTTTACAGTGTATCTCGAAGTCTTCCATCAGCAGTGATTGAAGTATCTGTACCT
GCCCCCACTCAGCATTTCGGTGCTTCCCTTTCACTGAAGTGAATACATGGTAGCAGGGTCTTTGTGTGCTGTGGATT
TTGTGGCTTCAATCTACGATGTTAAAACAAATTAAAAACACCTAAGTGACTACCACTTATTTCTAAATCCTCACTAT
TTTTTTGTTGCTGTTGTTCAGAAGTTGTTAGTGATTTGCTATCATATATTATAAGATTTTTAGGTGTCTTTTAATGA
TACTGTCTAAGAATAATGACGTATTGTGAAATTTGTTAATATATATAATACTTAAAAATATGTGAGCATGAAACTAT
GCACCTATAAATACTAAATATGAAATTTTACCATTTTGCGATGTGTTTTATTCACTTGTGTTTGTATATAAATGGTG
AGAATTAAAATAAAACGTTATCTCATTGCAAAAAT

C) *SAT1* 3’UTR

GGAGTGCTGCTGTAGATGACAACCTCCATTCTATTTTAGAATAAATTCCCAACTTCTCTTGCTTTCTATGCTGTT
TGTAGTGAAATAATAGAATGAGCACCCATTCCAAAGCTTTATTACCAGTGGCGTTGTTGCATGTTTGAAATGAGGTC
TGTTTAAAGTGGCAATCTCAGATGCAGTTTGGAGAGTCAGATCTTTCTCCTTGAATATCTTTCGATAAACAACAAGG
TGGTGTGATCTTAATATATTTGAAAAAAACTTCATTCTCGTGAGTCATTTAAATGTGTACAATGTACACACTGGTAC
TTAGAGTTTCTGTTTGATTCTTTTTTAATAAACTACTCTTTGATTTAA
