# Supplementary material for: Regulation of Parkinson’s disease-associated genes by Pumilio proteins and microRNAs in SH-SY5Y neuronal cells
Source: PLoS One. 2022 Sep 29;17(9):e0275235. doi: 10.1371/journal.pone.0275235 (PMC9522289; doi:10.1371/journal.pone.0275235)
Supplement: S4 Fig — (PDF) [file pone.0275235.s004.pdf]

Figure S4. Sequence conservation within putative canonical (cPRE) and non-canonical (ncPRE) PREs among mammals. Nucleotides that match the human sequence at each position are highlighted in gray. Indels are indicated by a “-“.

LRRK2 canonical PREs

	cPRE1	cPRE2	cPRE3
**Human**	**UGU-----AAAUA**	**UGUAAAUA**	**UGUAAAUA**
Chimp	UGU-----AAAUA	UGUAAAUA	UGUAAAUA
Rhesus	-------------	UGCAAAUA	UGUAAAUA
Squirrel	UGU-----AAAUA	UAUAAACA	UGUAAAUA
Mouse	UCUUUUUAAAAUA	UGUAAACU	UGUAAAUA
Rat	UAUUUUUAAAAUA	UGCAAACU	UGUAAACA
Rabbit	UGU-----AAAUA	UGCAGACA	UGUGGAUA
Pig	UGU-----AAAUA	UAUAAAUA	UGUAAAUA
Cow	UAU-----AAAUA	UGUAAAUA	UGUAAAUA
Cat	UGU-----AAAUA	UGUAAAC-	UGUGAAUA
Dog	UGU-----AAAUA	UGUAAACA	UGUAGAUA
Brown bat	UAU-----AAAUA	UGUAAAUA	UGUAAAUG
Elephant	UGU-----AAAUA	UGUAAAUA	UGUAAAUA

LRRK2 non-canonical PREs

	ncPRE1	ncPRE2	ncPRE3	ncPRE4	ncPRE5	ncPRE6
**Human**	**UGUAAAAAUA**	**UGUAUUUUAAA**	**UGUAUAAUUA**	**UGUAAUAAU**	**UGUAUUUUAAA**	**UGUAAUUA**
Chimp	UGUAAAAAUA	UGUAUUUUAAA	UGUAUAAUUA	UGUAAUAAU	UGUAUUUUAAA	UGUAAUUA
Rhesus	UGUAAAAAUA	UAUAUUUUAAA	UGUAUAAUUA	UGUAAUAAU	UGUAU------	CGUAAUUA
Squirrel	UGUAAAAAUA	UAUGUUCUAAG	UGUAUUAUUG	UGUAAAUAU	CAUUUUUCAAA	UGUCAACA
Mouse	UGUAAAAAUA	UAUGUUCUAAG	UAUAUAAUUG	UGUAGUAAU	-----------	UGAUAUU-
Rat	UGU-AAAAUA	UAUGUUUUAAA	UAUAGAAUUA	UGUAAUAAC	CACUCUUUAUA	--------
Rabbit	UGUAAAAAUA	UAUGUUUUAAG	UCUACAGUUG	UUUAAUAAU	CACUUUUCAAA	UAUAAUUA
Pig	UAU-AAAAUA	UAUAUUUUAAA	U-UAUAAUUG	UGUAGUAAU	UGU-UUUUCAA	UGUAGUUA
Cow	UAC-AAAAUU	UAUAUUUUAAA	U-UAUAAUUG	GGUAACCAU	CAU-UUUCAAA	UGUAGUUA
Cat	UGUAAAAAUA	-AUGUUUUAAA	UGUAUACUUG	GGUAACAAU	-----------	-------G
Dog	UGU-AAAAUA	UAUGUUUUAAA	UGUAUAAUUG	CAGAACAAA	-----------	-------A
Brown bat	UGUAAAAAUA	---------AA	UAUAACA---	GGUAACAAU	--U-UUUCAAA	UGUAGUU-
Elephant	UGU-AAAAUA	UAUGUUUAAAA	U-UAUCAUGC	UGCAACUUU	CAC-UUUCAAA	---AGUUA

SNCA

	ncPRE	cPRE
**Human**	**UGUACAAGU**	**UGUAUAUA**
Chimp	UGUACAAGU	UGUAUAUA
Rhesus	UGUACAAGU	UGUAUAUA
Squirrel	UGUACAAGU	UGUAUAUA
Mouse	CGUACAAGU	UGUAUAUA
Rat	CGUACAAGU	UGUAUAUA
Rabbit	UGUGCAAGU	UAUAUAUA
Pig	UGUCCAAGU	UGUAUAUA
Cow	UGUAUAAGU	UGUAUAUA
Cat	UGUACAAGU	UAUAUAUA
Dog	UGUACAAGU	UAUAUAUA
Brown bat	UGUACAAGU	UGUACAUA
Elephant	UCUACAAGU	UGAAUAUA

SAT1

	ncPRE1	ncPRE2
**Human**	**UGUAGUGAAAUA**	**UGUACAAU**
Chimp	UGUAGUGAAAUA	UGUACAAU
Rhesus	UGUAGUGAAAUA	UGUACAAU
Squirrel	UGUAGUAAAAUA	UGUACAAU
Mouse	UGUAGUGAAAUA	UGUACAAU
Rat	UGUAGUGAAAUA	UGUACAAU
Rabbit	UGUAGUGAAAUA	UGUACAAU
Pig	CGUAGUGGAAUA	UGUACAAU
Cow	UGUAGUGAAAUA	UGUACAAU
Cat	UGUAGUCGAAUA	UGUACAAU
Dog	UGUAGUCAAAUA	UGUACAAU
Brown bat	UGUAGUGAAAUA	UGUACAAU
Elephant	UGUAGUGGAAUA	UGUACAAU

For locations of PREs, see Fig S3.

Sequence alignments are based on output from gene searches in TargetScanHuman 8.0.

McGeary SE, Lin KS, Shi CY, Pham T, Bisaria N, Kelley GM, Bartel DP.The biochemical basis of microRNA targeting efficacy. Science Dec 5, (2019).

Agarwal V, Bell GW, Nam J, Bartel DP. Predicting effective microRNA target sites in mammalian mRNAs. eLife, 4:e05005, (2015). eLife Lens view.
