# Supplementary material for: Regulation of Parkinson’s disease-associated genes by Pumilio proteins and microRNAs in SH-SY5Y neuronal cells
Source: PLoS One. 2022 Sep 29;17(9):e0275235. doi: 10.1371/journal.pone.0275235 (PMC9522289; doi:10.1371/journal.pone.0275235)

Figure S5. Directed Acyclic Graphs for GO top 20.

A. Cellular Component DAG

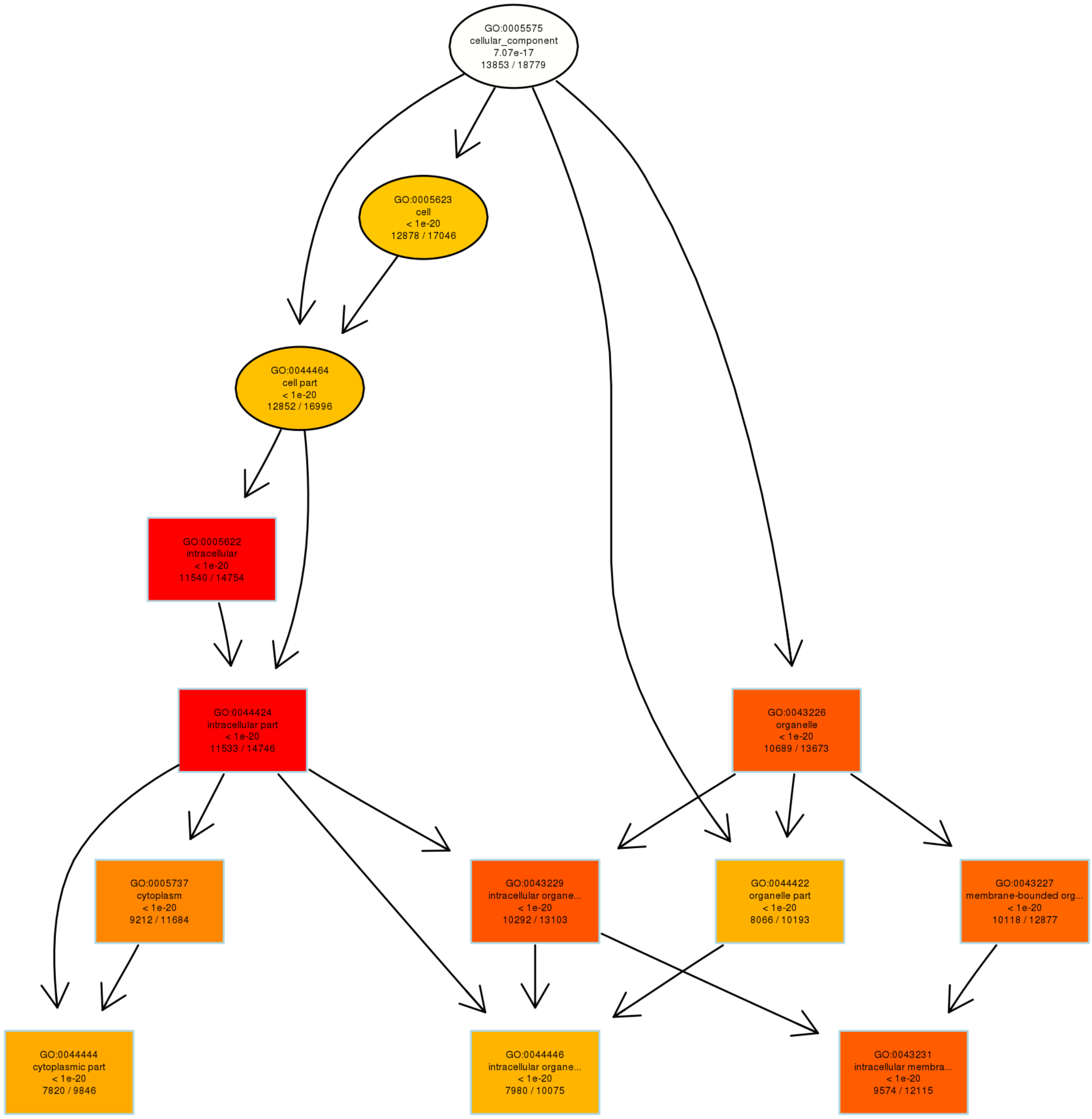

B. Biological Process DAG

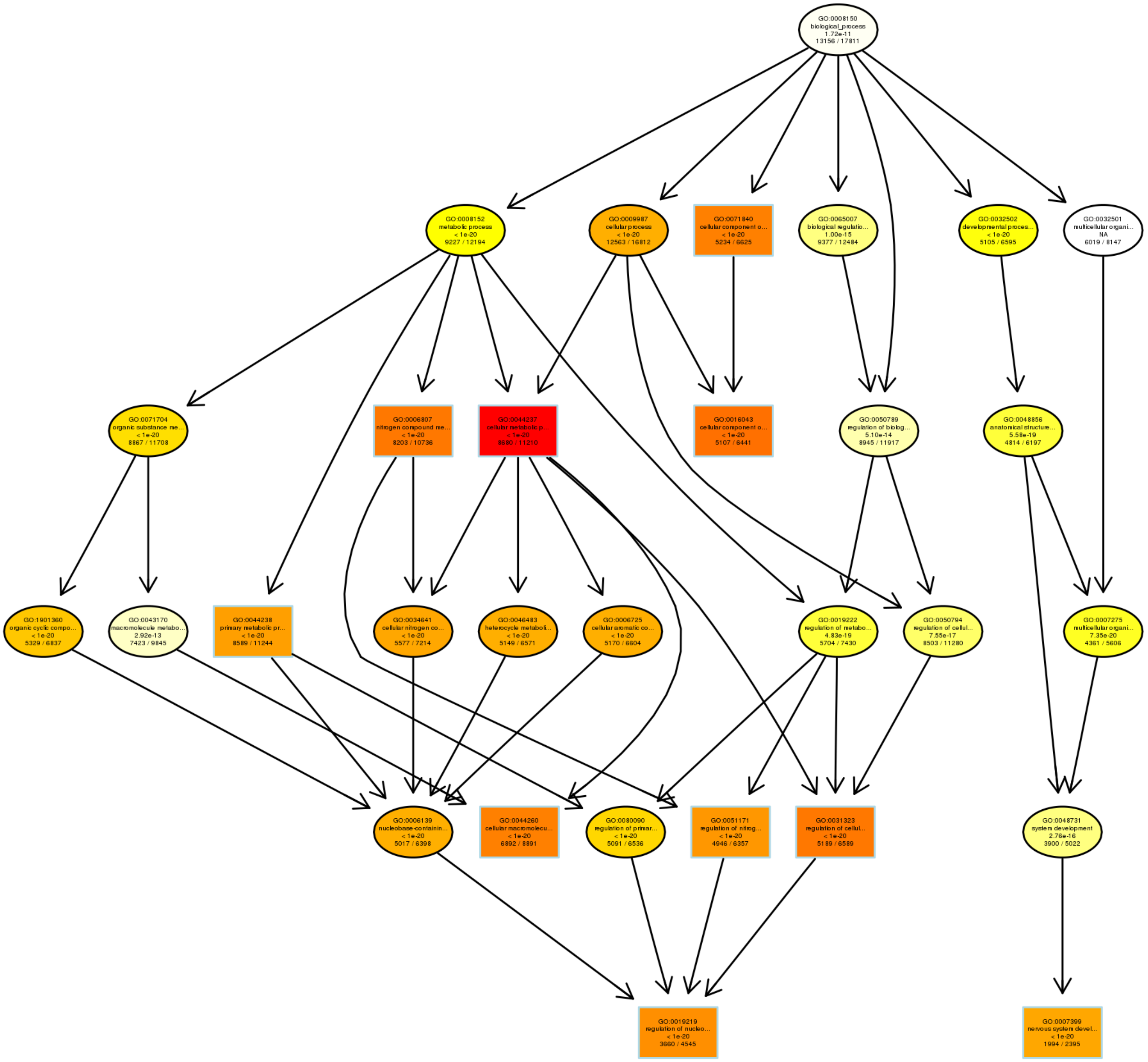

C. Molecular Function DAG

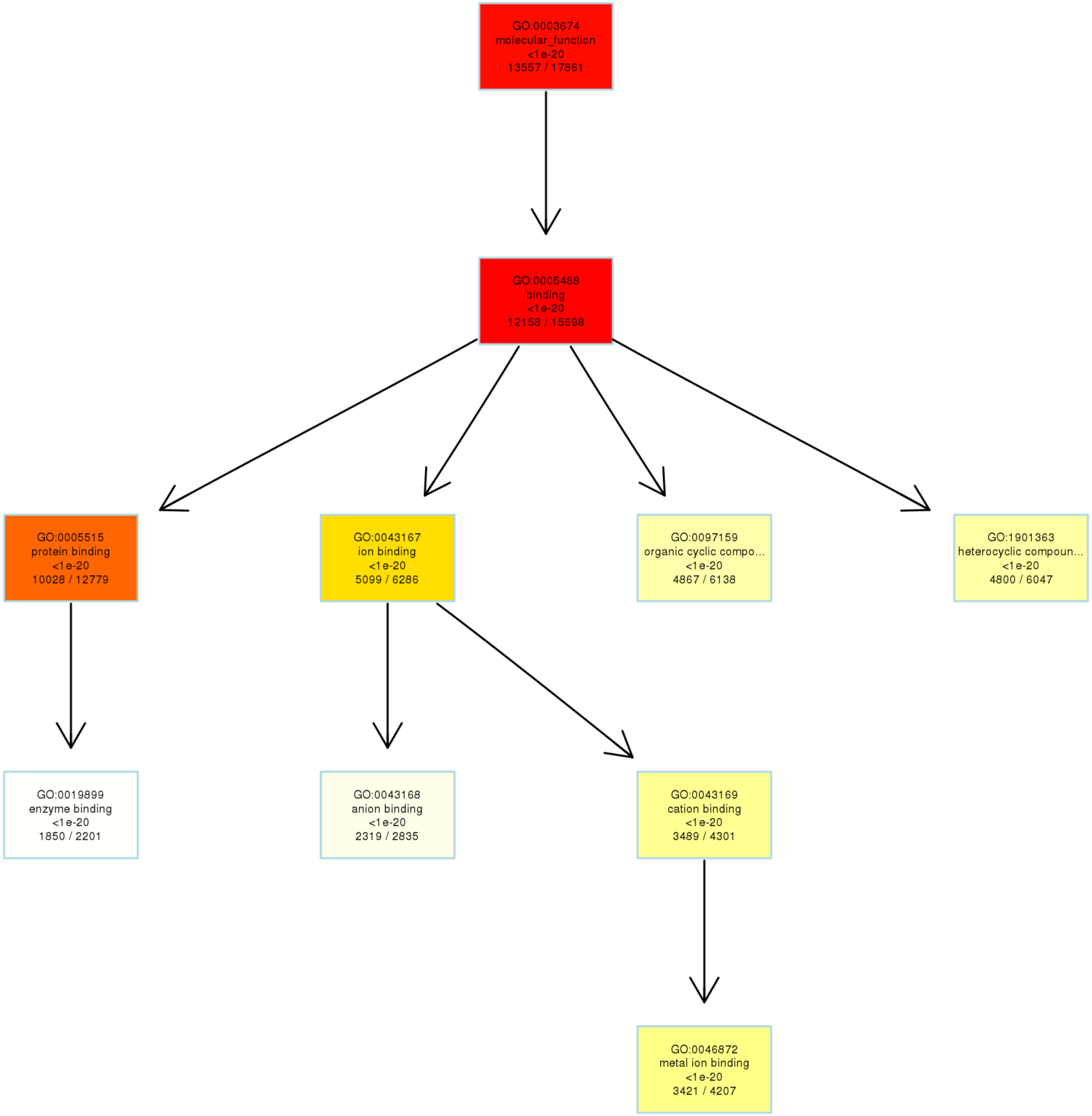

Supplement: S5 Fig — (PDF) [file pone.0275235.s005.pdf]
